# Nonselective β-Adrenergic Receptor Inhibitors Impair Hematopoietic Regeneration in Mice and Humans after Hematopoietic Cell Transplants

**DOI:** 10.1158/2159-8290.CD-24-0719

**Published:** 2024-12-30

**Authors:** Jinsuke Nishino, Wenhuo Hu, Ashwin Kishtagari, Bo Shen, Xiang Gao, Caroline M. Blackman, Adetola Kassim, Naimisha Marneni, Abhisar V. Cherukuri, Russell Vittrup, Fatma N. Kalkan, Rahul Shah, Chul Ahn, Ang Gao, Abeer Ahmedrabie, Robert H. Collins, Amer M. Zeidan, Aram Bidikian, Lohith Gowda, Brian C. Shaffer, Yazan F. Madanat, Zhiyu Zhao, Stephen S. Chung, Sean J. Morrison

**Affiliations:** 1 Children’s Research Institute and the Department of Pediatrics, University of Texas Southwestern Medical Center, Dallas, Texas.; 2 Department of Pathology, Center of Excellence for Leukemia Studies, St. Jude Children’s Research Hospital, Memphis, Tennessee.; 3 Division of Hematology/Oncology, Department of Internal Medicine, Vanderbilt University Medical Center, Nashville, Tennessee.; 4 National Institute of Biological Sciences, Beijing, China.; 5 Tsinghua Institute of Multidisciplinary Biomedical Research, Tsinghua University, Beijing, China.; 6 Division of Hematology/Oncology, Department of Internal Medicine, University of Texas Southwestern Medical Center, Dallas, Texas.; 7 Department of Population and Data Sciences, University of Texas Southwestern Medical Center, Dallas, Texas.; 8 Section of Hematology, Department of Internal Medicine, Yale University School of Medicine and Yale Cancer Center, New Haven, Connecticut.; 9 Department of Medicine, Adult Bone Marrow Transplantation Service, Memorial Sloan Kettering Cancer Center, New York, New York.; 10 Howard Hughes Medical Institute, UT Southwestern Medical Center, Dallas, Texas.

## Abstract

Peripheral nerves promote mouse bone marrow regeneration by activating β2- and β3-adrenergic receptor signaling, raising the possibility that nonselective β-blockers could inhibit engraftment after hematopoietic cell transplants (HCT). We observed no effect of β-blockers on steady-state mouse hematopoiesis. However, mice treated with a nonselective β-blocker (carvedilol), but not a β1-selective inhibitor (metoprolol), exhibited impaired hematopoietic regeneration after syngeneic or allogeneic HCTs. At two institutions, patients who received nonselective, but not β1-selective, β-blockers after allogeneic HCT exhibited delayed platelet engraftment and reduced survival. This was particularly observed in patients who received posttransplant chemotherapy for graft-versus-host disease prophylaxis, which also accentuated the inhibitory effect of carvedilol on engraftment in mice. In patients who received autologous HCTs, nonselective β-blockers were associated with little or no delay in engraftment. The inhibitory effect of nonselective β-blockers after allogeneic HCT was overcome by transplanting larger doses of hematopoietic cells.

**Significance:**

Patients who receive allogeneic HCTs followed by posttransplant chemotherapy for graft-versus-host disease prophylaxis may be at risk of delayed engraftment and increased mortality if administered nonselective β-blockers after transplantation. Transient discontinuation of nonselective β-blockers or transitioning to β1-selective inhibitors after HCT may accelerate engraftment and improve clinical outcomes.

*See related commentary by Bhatia, p. 666*

## Teaser

Analyses of mice and patients who underwent hematopoietic cell transplants reveal that use of non-selective β-blockers, but not β1-selective inhibitors, impaired hematopoietic regeneration and worsened survival.

## Introduction

The bone marrow (BM) contains peripheral nerves, including sympathetic (1), parasympathetic (2), and sensory (3, 4) nerve fibers. Sympathetic denervation by systemic 6-hydroxydopamine treatment does not affect hematopoietic stem cell (HSC) frequency or hematopoiesis under steady-state conditions (5), but peripheral nerves regulate the circadian mobilization of hematopoietic stem/progenitor cells into the blood (1, 3, 6, 7) and the regeneration of hematopoiesis after irradiation or chemotherapy (2, 5). Peripheral nerves promote hematopoietic regeneration by activating β2- and β3-adrenergic receptor signaling (5, 8, 9) in leptin receptor-expressing (LepR^+^) stromal cells in adult mouse BM (10).

LepR^+^ stromal cells in adult mouse BM are a critical source of growth factors that promote the maintenance of hematopoietic stem and progenitor cells (11–15). These cells have also been referred to as CXCl12-abundant reticular cells (16, 17). LepR^+^ cells promote the maintenance of HSCs and early restricted progenitors by synthesizing stem cell factor (SCF; refs. 11–13) and CXCL12 (14, 15), among other factors (18–20). The analyses of SCF (11) and CXCL12 (14–17) reporter mice, as well as single-cell RNA sequencing (21–23), have shown that LepR^+^ cells are the main source of these factors in adult BM. LepR^+^ cells also promote vascular regeneration in the BM after irradiation or chemotherapy by producing Angiopoietin-1 (24) and VEGF-C (25). Finally, LepR^+^ cells include the skeletal stem and progenitor cells that form the osteoblasts and adipocytes that arise in adult BM (16, 26, 27). These adipocytes also promote the regeneration of HSCs and hematopoiesis after myeloablation by synthesizing SCF (28).

We recently found that peripheral nerve maintenance in adult mouse BM depends upon nerve growth factor (NGF) produced by LepR^+^ cells (10). NGF deletion from LepR^+^ cells leads to the loss of nerves from adult BM. Consistent with prior studies (2, 5), this did not affect hematopoiesis under steady-state conditions but profoundly impaired hematopoietic and vascular regeneration in the BM after irradiation or chemotherapy. Nerves promote BM regeneration by releasing catecholamines, which activate β2- and β3-adrenergic receptor signaling in LepR^+^ cells (10). β2/β3 signaling increases the production of multiple growth factors by LepR^+^ cells that are necessary for hematopoietic and vascular regeneration, including SCF, VEGF, and Angiopoietin-2. Deletion of β2- and β3-adrenergic receptors from LepR^+^ cells phenocopies the effects of nerve loss, with no effect on steady-state hematopoiesis but reduced growth factor expression as well as hematopoietic and vascular regeneration after irradiation or chemotherapy. Nerves and LepR^+^ cells thus promote BM regeneration through a reciprocal relationship in which LepR^+^ cells sustain nerves by synthesizing NGF and nerves promote regeneration by activating β2/β3 signaling in LepR^+^ cells.

These data raised the question of whether patients who happen to be on a β-blocker when receiving a hematopoietic cell transplant (HCT) exhibit delayed hematopoietic regeneration. Delayed hematopoietic regeneration after allogeneic HCT in patients is associated with significantly worse clinical outcomes (29, 30). We observed no effect of β-blockers on steady-state hematopoiesis in mice, consistent with the studies described above (10). However, after syngeneic or allogeneic BM transplants in mice, treatment with a nonselective β-blocker (with activity against β2/β3), but not a β1-selective inhibitor, impaired hematopoietic regeneration. A retrospective analysis of autologous and allogeneic transplant recipients revealed similar effects after allogeneic HCT in humans, particularly in patients who received posttransplant chemotherapy for graft-versus-host disease (GvHD) prophylaxis.

## Results

### Nonselective β-Blockers Impaired Hematopoietic Regeneration after HCT in Mice

To test if β-blockers alter hematopoiesis under steady-state conditions, we treated 8- to 12-week-old C57BL/Ka mice with carvedilol (a nonselective β-blocker; ref. 31), metoprolol (a β1-selective inhibitor; ref. 31), or vehicle for 21 days and then analyzed hematopoiesis. Neither carvedilol-treated nor metoprolol-treated mice significantly differed from vehicle-treated controls in terms of white blood cell (WBC), red blood cell (RBC), or platelet (PLT) counts, BM or spleen cellularity, or the frequencies of CD150^+^CD48^−^Lineage^−^Sca-1^+^c-kit^+^ HSCs, CD150^−^CD48^−^Lineage^−^Sca-1^+^c-kit^+^ multipotent progenitors (MPP), or Lineage^−^Sca-1^+^c-kit^+^ (LSK) stem/progenitor cells in the BM or spleen (Supplementary Fig. S1A–S1K for carvedilol and Supplementary Fig. S1L–S1V for metoprolol). Representative flow cytometry gates are provided in Supplementary Fig. S1W. We did not detect any effect of selective or nonselective β-blockers on hematopoiesis in adult mice under steady-state conditions.

**Figure 1.**
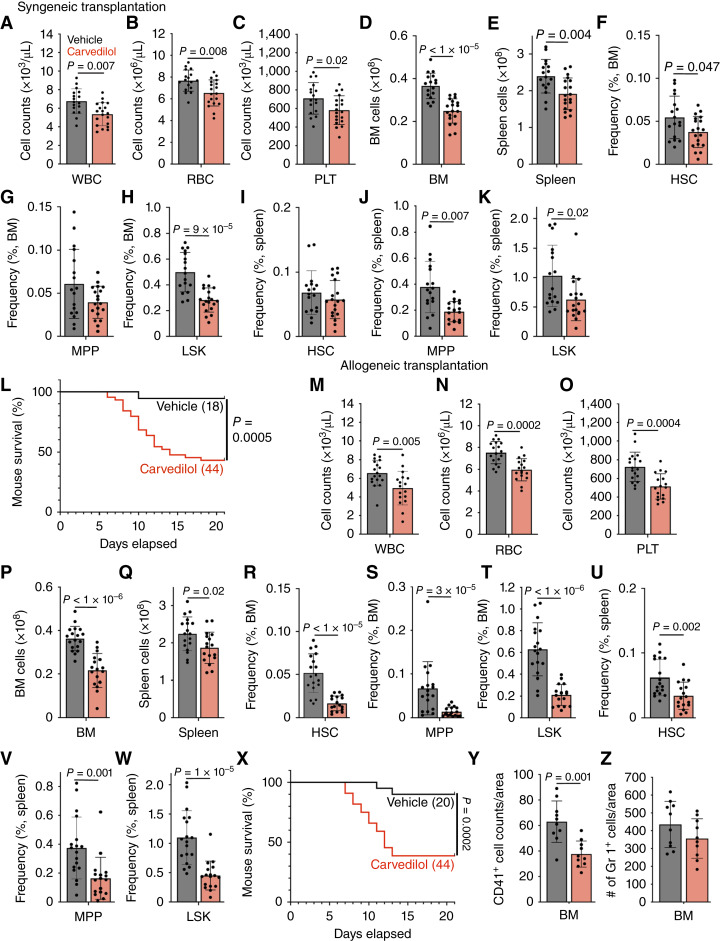
Carvedilol treatment impairs hematopoietic regeneration in mice after syngeneic and allogeneic BM transplants. Mice were treated with nonselective β-blocker, carvedilol, or vehicle control for 7 days before BM transplantation and 21 days after transplantation. Each panel shows data from four independent experiments, and each dot represents a different mouse. All data represent mean ± SD. **A–L,** 6 × 10^5^ C57BL/Ka BM cells were syngeneically transplanted into irradiated C57BL/Ka-Thy-1.2 recipients. At 21 days after transplantation, we analyzed WBC (**A**), RBC (**B**), and PLT (**C**) counts; BM (**D**) and spleen (**E**) cellularity; and the frequencies of HSCs, MPPs, and LSK cells in the BM (**F–H**) and spleen (**I–K**) of carvedilol-treated (red, *n* = 19) and vehicle-treated (black, *n* = 17) mice. **L,** Survival of carvedilol-treated (red, *n* = 44) and vehicle-treated (black, *n* = 18) mice over time after syngeneic transplantation. **M**–**X,** 6 × 10^5^ T cell–depleted LP/J BM cells were allogeneically transplanted into irradiated C57BL/Ka-Thy-1.2 recipients. At 21 days after transplantation, we analyzed WBC (**M**), RBC (**N**), and PLT (**O**) counts, as well as BM (**P**) and spleen (**Q**) cellularity and the frequencies of HSCs, MPPs, and LSK cells in the BM (**R–T**) and spleen (**U–W**) of carvedilol-treated (red, *n* = 17) and vehicle-treated (black, *n* = 18) mice. **X,** Survival of carvedilol-treated (red, *n* = 44) and vehicle-treated (black, *n* = 20) mice over time after allogeneic transplantation. **Y** and **Z,** The number of CD41^+^ megakaryocytes (**Y**) or Gr1^+^ myeloid cells (**Z**) per field of view was counted in BM sections from carvedilol-treated (red, *n* = 10) and vehicle-treated (black, *n* = 10) mice at 13 days after transplantation. The statistical significance of differences among treatments were assessed using the Student *t* tests followed by Holm–Sidak multiple comparisons adjustments (**A–C** and **M–T**), matched samples two-way ANOVAs followed by Holm–Sidak multiple comparisons adjustments (**D**, **E**, **I–K**, **Y**, and **Z**), Student or Welch’s *t* tests followed by Holm–Sidak multiple comparisons adjustments (**F–H**), Mann–Whitney tests followed by Holm–Sidak multiple comparisons adjustments (**U–W**), or log-rank Mantel–Cox tests (**L** and **X**). All statistical tests were two sided.

To test if carvedilol had any effect on the engraftment of BM cells after syngeneic transplantation, we treated C57BL/Ka mice with carvedilol or vehicle for 7 days before and 21 days after irradiation and transplantation of 6 × 10^5^ C57BL/Ka BM cells. At 21 days after transplantation, the carvedilol-treated mice exhibited significantly lower WBC, RBC, and PLT counts (Fig. 1A–C), as well as reduced BM and spleen cellularity (Fig. 1D and E) and lower frequencies of HSCs and LSK cells in the BM and MPPs and LSK cells in the spleen (Fig. 1F–K). Mice treated with carvedilol exhibited significantly worse survival after transplantation: 94% of vehicle-treated mice and 43% of carvedilol-treated mice survived after transplantation (Fig. 1L). Treatment of mice with a nonselective β-blocker thus impaired hematopoietic regeneration and survival after syngeneic BM transplantation.

We did a similar experiment using metoprolol instead of carvedilol. Metoprolol-treated and vehicle-treated mice did not significantly differ in terms of blood cell counts, BM or spleen cellularity, or the frequencies of HSCs, MPPs, or LSK cells in the BM or spleen at 21 days after bone marrow transplant (BMT) (Supplementary Fig. S2A–S2K). Metoprolol treatment also did not significantly affect survival (Supplementary Fig. S2L). Thus, β1-selective inhibition did not affect hematopoietic regeneration or survival after syngeneic BM transplantation in mice.

To test if carvedilol impaired engraftment after allogeneic BM transplantation, we treated C57BL/Ka mice with carvedilol or vehicle for 7 days before and 21 days after irradiation and transplantation of 6 × 10^5^ T cell–depleted LP/J BM cells. LP/J mice have the same MHC alleles as C57BL/Ka mice but differ in terms of minor histocompatibility alleles (32). The carvedilol-treated mice exhibited significantly lower WBC, RBC, and PLT counts (Fig. 1M–O) as well as reduced BM and spleen cellularity (Fig. 1P and Q) and lower frequencies of HSCs, MPPs, and LSK cells in the BM and spleen (Fig. 1R–W). The carvedilol-treated mice also exhibited significantly worse survival: 90% of vehicle-treated and 39% of carvedilol-treated mice survived 21 days after transplantation (Fig. 1X). The number of CD41^+^ megakaryocytes per field of view was significantly reduced in the BM sections from carvedilol-treated as compared with untreated mice at 13 days after transplantation (Fig. 1Y). Gr1^+^ myeloid cells also exhibited a trend toward reduced numbers in BM sections from carvedilol-treated mice but the difference was not statistically significant (Fig. 1Z). Treatment with a nonselective β-blocker thus impaired hematopoietic regeneration and survival in mice after syngeneic and allogeneic BM transplants.

In contrast to these results with carvedilol, metoprolol-treated and vehicle-treated mice did not significantly differ in terms of blood cell counts, BM or spleen cellularity, survival, or the frequencies of HSCs, MPPs, or LSK cells in the BM or spleen at 21 days after transplantation of T cell–depleted LP/J BM cells into C57BL/Ka mice (Supplementary Fig. S2M–S2X). These results with pharmacological inhibitors are consistent with our genetic analysis, which showed that β2- and β3-adrenergic receptors are necessary in LepR^+^ cells for BM regeneration after irradiation or chemotherapy, whereas β1-adrenergic receptors are not (10).

### Nonselective β-Blockers Impaired Hematopoietic Regeneration after Allogeneic HCT

To determine if nonselective β-blockers impair hematopoietic regeneration in humans, we evaluated patients who received a HCT. We first evaluated β-blocker use in all patients at University of Texas Southwestern Medical Center (UTSW) who underwent autologous (*n* = 871; Supplementary Table S1) or allogeneic (*n* = 311; Supplementary Table S2) HCT between January 2013 and January 2023. All patients who received autologous HCTs and 87.4% of patients who received allogeneic HCTs received peripheral blood stem cell grafts. The remaining 12.6% receiving unstimulated BM grafts. Charts were reviewed to confirm β-blocker use within 15 days after transplantation. Carvedilol was the most commonly used nonselective β-blocker (*n* = 74), but some patients received propranolol (*n* = 5) or labetalol (*n* = 5). Metoprolol was the most commonly used β1-selective inhibitor (*n* = 128), but some patients received atenolol (*n* = 11) or bisoprolol (*n* = 7). We then assessed whether β-blocker use was associated with impaired hematopoietic regeneration as measured by increased time to neutrophil or PLT engraftment using standard clinical criteria (see “Methods”; ref. 33).

**Figure 2.**
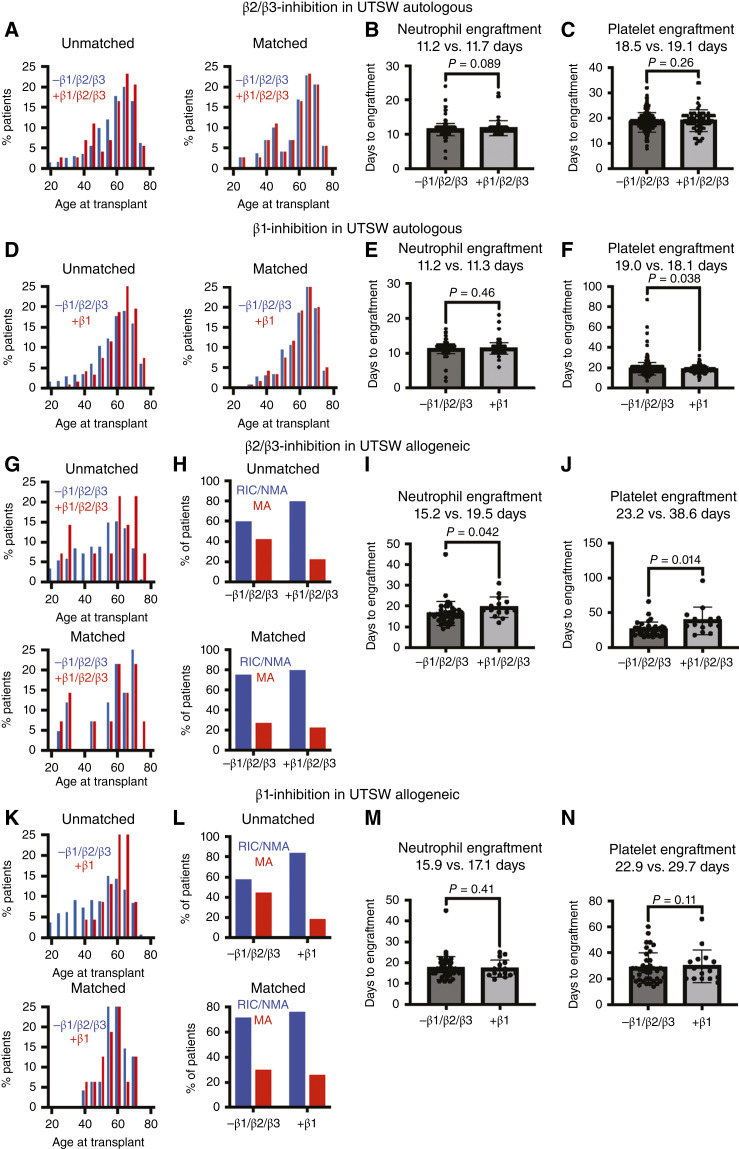
Nonselective β-adrenergic receptor inhibitors impair hematopoietic regeneration in patients after autologous and allogeneic transplantation. **A,** Propensity matching for age in recipients who received autologous transplant at UTSW who were (*n* = 73), or were not (*n* = 798), treated with nonselective β-blockers. The time to neutrophil (**B**) and platelet (**C**) engraftment in matched groups (*n* = 73 and 219 after 1:3 matching) is shown. **D,** Propensity matching for age in patients who received autologous transplant at UTSW who were (*n* = 123), or were not (*n* = 675), treated with β1-selective inhibitors. The time to neutrophil (**E**) and platelet (**F**) engraftment in matched groups (*n* = 120 and 360 after 1:3 matching). Propensity matching for age (**G**) and conditioning regimen (**H**) in patients who received allogeneic transplant at UTSW who were (*n* = 14), or were not (*n* = 297), treated with nonselective β-adrenergic receptor inhibitors (RIC, NMA, vs. myeloablative conditioning). The time to neutrophil (**I**) and platelet (**J**) engraftment in matched groups (*n* = 14 and 42 after 1:3 matching). Propensity matching for age (**K**) and conditioning regimen (**L**) in patients receiving allogeneic transplant at UTSW who were treated with β1-selective inhibitors (*n* = 23) vs. no β-blocker (*n* = 274). The time to neutrophil (**M**) and platelet (**N**) engraftment in matched (*n* = 16 and 48 after 1:3 matching). The statistical significance of differences among groups was assessed using Student *t* tests. All data represent mean ± SD.

We first evaluated autologous HCT recipients. Because patients on nonselective β-blockers (*n* = 73; mean age, 58.7 years) were slightly older than those not on nonselective β-blockers (*n* = 798; mean age, 57.9 years), propensity score matching was performed to derive cohorts of patients (1:3 matching, *n* = 73 and 219) matched for age (mean age, 58.7 vs. 58.6 years; Fig. 2A). In the matched cohorts, patients on nonselective β-blockers, compared with those not on nonselective β-blockers, exhibited a trend toward prolonged time to neutrophil engraftment (11.7 vs. 11.2 days, *P* = 0.089; Fig. 2B) but not PLT engraftment (*P* = 0.26; Fig. 2C). The use of nonselective β-blockers by autologous HCT recipients at UTSW was thus associated with a trend toward modestly delayed neutrophil engraftment.

We next compared patients on β1-selective inhibitors (*n* = 123) with those not on β-blockers (*n* = 675). Since patients on β1 inhibitors were older than those not on β-blockers (mean age, 61.1 vs. 57.0 years), propensity score matching was again performed to derive cohorts of patients (1:3 matching, *n* = 120 and 360) matched for age (mean age, 60.7 vs. 60.5 years; Fig. 2D). In these matched cohorts, patients on β1-selective inhibitors and patients not on β-blockers exhibited no difference in time to neutrophil engraftment (*P* = 0.46; Fig. 2E) and a somewhat shorter time to PLT engraftment (18.1 vs. 19.0 days, *P* = 0.038; Fig. 2F). Thus, consistent with our results in mice, the use of β1-selective inhibitors was not associated with impaired hematopoietic regeneration in patients after autologous HCT.

We also evaluated the effect of β-blockers on neutrophil and PLT regeneration after allogeneic HCT. Patients on nonselective β-blockers (*n* = 14), as compared with those not on nonselective β-blockers (*n* = 297), were older (mean age, 56.4 vs. 50.3 years) and more likely to have received reduced-intensity conditioning or non–myeloablative conditioning (RIC/NMA, 78.6% vs. 58.6%). Thus, propensity score matching was performed to derive cohorts of patients (1:3 matching, *n* = 14 and 42, respectively) matched for age (mean age, 56.4 vs. 57.1 years) and conditioning intensity (78.6% vs. 73.8% RIC/NMA; Fig. 2G and H). In these matched cohorts, patients on nonselective β-blockers, compared with those not on nonselective β-blockers, exhibited a prolonged time to neutrophil (19.5 vs. 15.2 days, *P* = 0.042; Fig. 2I) and PLT engraftment (38.6 vs. 23.2 days, *P* = 0.014; Fig. 2J). These prolonged times to engraftment would be highly clinically significant, as they would prolong hospitalization and increase the risk of infection and bleeding.

We also compared patients on β1-selective inhibitors (*n* = 23) with those not on β-blockers (*n* = 274). Patients on β1 inhibitors were older (mean age, 60.3 vs. 49.5 years) and more likely to have received RIC/NMA (82.6% vs. 56.6%) than those not on β-blockers, so propensity score matching was performed to derive cohorts of patients (1:3 matching, *n* = 16 and 48) matched for age (mean age, 58.3 vs. 58.3 years) and conditioning (75% vs. 70.8% RIC/NMA; Fig. 2K and L). In these matched cohorts, patients on β1-selective inhibitors did not significantly differ from patients not on β-blockers in the time to neutrophil (*P* = 0.41; Fig. 2M) or PLT engraftment (*P* = 0.11; Fig. 2N). Thus, the use of nonselective β-blockers, but not β1-selective inhibitors, by patients who underwent allogeneic HCT was associated with substantially delayed neutrophil and PLT engraftment.

We next evaluated the effect of β-blockers on hematopoietic regeneration in two external validation cohorts of patients who underwent autologous or allogeneic HCT at Vanderbilt University Medical Center (*n* = 1,109 and 549, respectively: Supplementary Tables S3 and S4). When compared with patients who underwent allogeneic HCT at UTSW, patients who underwent allogeneic HCT at Vanderbilt exhibited more heterogeneity in covariates known to affect engraftment, such as cell dose and receipt of posttransplant myelosuppressive chemotherapy. For example, the SD of cell dose was 1.9 × 10^6^ CD34^+^ cells/kg in the patients from Vanderbilt and 0.98 × 10^6^ CD34^+^ cells/kg in those from UTSW. Only 47% of patients from Vanderbilt received posttransplant myelosuppressive chemotherapy, as compared with 97% of patients from UTSW. Nonetheless, we began our analysis of these cohorts by performing the same propensity score matching analysis that we performed on the UTSW cohorts, acknowledging that it may be inadequate to account for these covariates.

We first evaluated autologous transplant recipients who were (*n* = 74) or were not (*n* = 1,037) treated with nonselective β-blockers. After propensity score matching to derive cohorts of patients (1:3 matching, *n* = 74 and 222) matched for age (mean age, 59.6 vs. 59.5 years; Supplementary Fig. S3A), we found that patients on nonselective β-blockers, compared with those not on nonselective β-blockers, exhibited no significant difference in the time to neutrophil or PLT engraftment (Supplementary Fig. S3B and S3C). We next evaluated patients with autologous transplant treated with β1-selective inhibitors (*n* = 210) or not on β-blockers (*n* = 827). After propensity score matching to derive cohorts of patients (1:3 matching, *n* = 154 and 462) matched for age (mean age, 59.3 vs. 59.2 years; Supplementary Fig. S3D), we found that patients on β1-selective inhibitors, compared with those not on β-blockers, exhibited no difference in time to neutrophil or PLT engraftment (Supplementary Fig. S3E and S3F). Thus, we did not observe any significant effects of nonselective β-blockers or β1-selective inhibitors on hematopoietic regeneration in patients who received autologous HCT at Vanderbilt.

We evaluated recipients with allogeneic transplant who were (*n* = 33) or were not (*n* = 543) treated with nonselective β-blockers. After propensity score matching to derive cohorts of patients (1:3 matching, *n* = 33 and 99) matched for age (mean 56.9 vs. 56.7) and conditioning regimen (66.6% vs. 65.7% RIC/NMA; Supplementary Fig. S3G and S3H), we found that patients on nonselective β-blockers, compared with those not on nonselective β-blockers, exhibited no significant difference in the time to neutrophil or PLT engraftment (Supplementary Fig. S3I and S3J). We next evaluated patients who received allogeneic transplant who were (*n* = 93) or were not (*n* = 450) treated with β1-selective inhibitors. After propensity score matching to derive cohorts of patients (1:3 matching, *n* = 66 and 198) matched for age (mean age, 56.1 vs. 55.8 years) and conditioning regimen (60.6% vs. 60.6% RIC/NMA; Supplementary Fig. S3K and S3L), we found that patients on β1-selective inhibitors, compared with those not on β-blockers, exhibited no difference in the time to neutrophil or PLT engraftment (Supplementary Fig. S3M and S3N). Thus, in propensity-matched cohorts of patients undergoing allogeneic HCT at Vanderbilt, we did not observe any significant effects of nonselective β-blockers or β1-selective inhibitors on hematopoietic regeneration. We hypothesized that the apparent lack of effect of nonselective β-blockers, in contrast to our findings in patients receiving allogeneic HCT at UTSW, may have been due to the increased heterogeneity among patients at Vanderbilt in terms of covariates that affect engraftment but which were not accounted for in the propensity-matched analysis.

### Multivariate Analysis of the Effect of Nonselective β-Blockers on Hematopoietic Regeneration after HCT

To determine if nonselective β-blockers represent an independent risk factor for delayed engraftment after HCT, in which accounting for covariates could also impact engraftment, we performed a multivariate analysis. We first evaluated patients at UTSW undergoing autologous and allogeneic HCT. To identify covariates associated with differences in engraftment, we first performed single-variable logistic regression analyses, evaluating a number of parameters that influence clinical outcomes after HCT (Supplementary Figs. S4A, S4B, and S5). From these analyses, we derived a risk factor coefficient (*B*), which reflects the number of additional days required for neutrophil or PLT engraftment per unit of each predictive variable. Increased age was associated with delayed neutrophil engraftment in patients who received autologous and allogeneic HCT, as well as delayed PLT engraftment in patients who received allogeneic HCT (Supplementary Figs. S4A, S5A, and S5B). Delayed neutrophil and PLT engraftment after allogeneic HCT was associated with factors known to have such effects, including RIC/NMA, underlying myeloid disease, and the use of a mismatched donor (Supplementary Fig. S5A and S5B; ref. 34).

**Figure 3.**
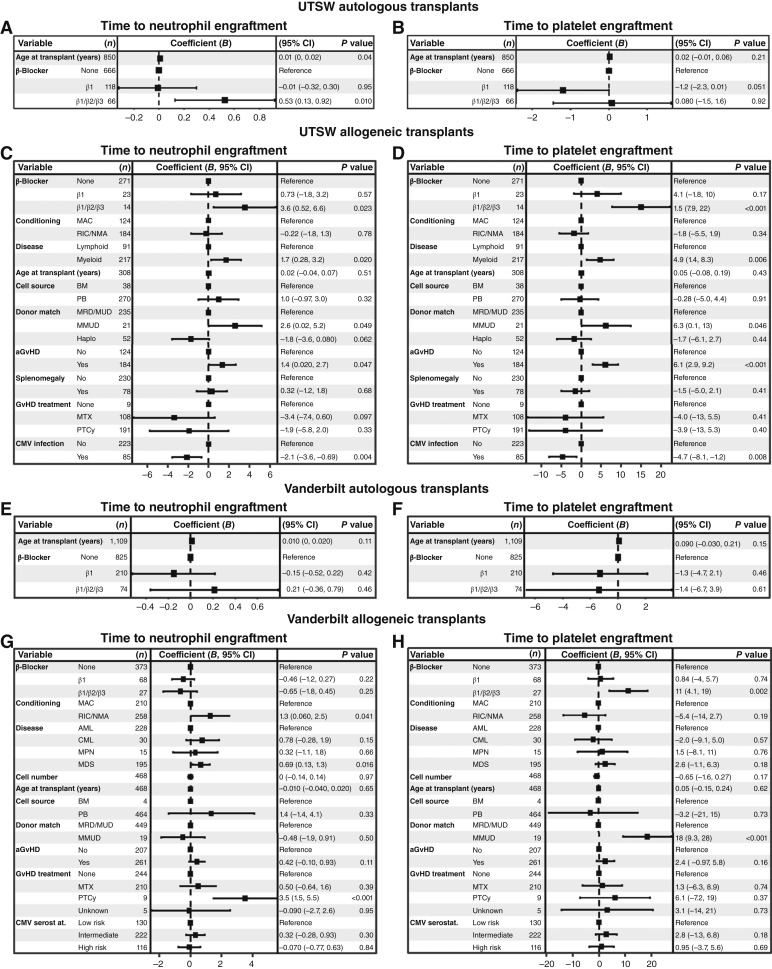
The use of nonselective β-blockers is an independent risk factor for delayed hematopoietic regeneration after allogeneic transplantation. The risk factor coefficients (*B*) for increased time to neutrophil (**A**) and platelet (**B**) engraftment among patients undergoing autologous HCT at UTSW, neutrophil (**C**) and PLT (**D**) engraftment among patients undergoing allogeneic HCT at UTSW, neutrophil (**E**) and PLT (**F**) engraftment among patients undergoing autologous HCT at Vanderbilt, and neutrophil (**G**) and PLT (**H**) engraftment among patients undergoing allogeneic HCT at Vanderbilt. A generalized linear model was used, with covariates including age, β1-selective inhibitor use, and nonselective β-blocker use for all cohorts. For patients receiving allogeneic HCT, other covariates included conditioning regimen [myeloablative conditioning (MAC) or RIC/NMA]; underlying disease [for UTSW lymphoid or myeloid, for Vanderbilt acute myeloid leukemia (AML), chronic myeloid leukemia (CML), myeloproliferative neoplasm (MPN), or myelodysplastic syndrome (MDS)]; cell dose (CD34^+^ cells/kg); cell source [BM or peripheral blood (PB)]; donor matching [matched related/matched unrelated donor (MRD/MUD), mismatched unrelated donor (MMUD), or haploidentical (Haplo)]; acute GvHD; and myelosuppressive GvHD treatment [methotrexate (MTX) or posttransplant Cytoxan (PTCy)]. Patients at UTSW also included splenomegaly and CMV infection as covariates, and patients at Vanderbilt included CMV serostatus (low risk = donor−/recipient−; intermediate risk = donor+/recipient+ or −; high risk = donor−/recipient+). *B* reflects the number of additional days required for neutrophil or PLT engraftment per unit of each predictive variable, with units being per year for age and binary (yes/no) for all other variables. *B* ± 95% confidence interval is shown. The dashed vertical line represents *B* of 0.

We then performed a multivariate analysis using a generalized linear model to evaluate the effects of β1-selective inhibitors and nonselective β-blockers on the time to neutrophil and PLT engraftment, taking into account the age for both cohorts. In patients undergoing autologous HCT at UTSW, the use of nonselective β-blockers, but not β1-selective inhibitors, was associated with increased time to neutrophil engraftment (*B* = 0.53 days, *P* = 0.010 vs. *B* = −0.010, *P* = 0.95; Fig. 3A) but not PLT engraftment (*B* = 0.080, *P* = 0.92 vs. *B* = −1.2, *P* = 0.051; Fig. 3B).

For patients undergoing allogeneic HCT at UTSW, additional covariates were included, based on their significance in the single-variable analyses or known potential to influence engraftment, including conditioning regimen, underlying disease, age, cell source, donor mismatch, acute GvHD, and cytomegalovirus (CMV) infection (34), as well as splenomegaly (35) and myelosuppressive GvHD prophylaxis (36). In patients undergoing allogeneic HCT at UTSW, the use of nonselective β-blockers, but not β1-selective inhibitors, was associated with increased time to neutrophil (*B* = 3.6 days, *P* = 0.023 vs. *B* = 0.73, *P* = 0.57; Fig. 3C) and PLT engraftment (*B* = 15 days, *P* < 0.001 vs. *B* = 4.1, *P* = 0.17; Fig. 3D). We also observed known associations between delayed neutrophil and PLT engraftment and underlying myeloid disease, donor mismatch (34), and acute GvHD (37), but all of these effects were smaller in magnitude than the effect of nonselective β-blockers. These results in patients undergoing HCT at UTSW corroborate the results of the propensity-matched analyses, demonstrating that the use of nonselective β-blockers is independently associated with impaired hematopoietic regeneration after HCT.

We next performed multivariate analyses of patients at Vanderbilt who underwent autologous and allogeneic HCT. We first performed single-variable logistic regression analyses. In both of these cohorts, there was no significant association between age and the time to engraftment (Supplementary Figs. S6A, S6B, S7A, and S7B). In the Vanderbilt allogeneic HCT cohort, delayed engraftment of neutrophils and PLTs was associated with known factors such as peripheral blood graft source (34) and receipt of posttransplant chemotherapy (Supplementary Fig. S7A and S7B; ref. 36). This cohort only included patients with underlying myeloid disease, and we observed a known association between underlying chronic myeloid leukemia and myelodysplastic syndromes and delayed neutrophil engraftment (Supplementary Fig. S7A; refs. 34, 38). Acute GvHD was associated with delayed neutrophil engraftment, as has been shown in Supplementary Fig. S7A (37). Delayed engraftment of PLTs was associated with known factors such as donor mismatch (34) and GvHD prophylaxis with methotrexate (Supplementary Fig. S7B; ref. 39). RIC/NMA was associated with accelerated PLT engraftment, as has been described (40).

We next performed a multivariate analysis using a generalized linear model to evaluate the effects of nonselective β-blockers and β1-selective inhibitors on neutrophil and PLT engraftment in patients undergoing HCT at Vanderbilt, taking into account the age for both cohorts (Fig. 3E–H). For recipients of allogeneic HCT, additional covariates were included on the basis of their significance in the single-variable analyses or known potential to influence engraftment, including conditioning regimen, underlying disease, cell dose, age, cell source, donor mismatch, and CMV serostatus (as a surrogate for infection risk; ref. 34), as well as acute GvHD (37) and myelosuppressive GvHD prophylaxis (36). In patients who received autologous HCT, we did not observe any effect of nonselective β-blockers or β1-selective inhibitors on engraftment of neutrophils or PLTs (Fig. 3E and F). In patients undergoing allogeneic HCT at Vanderbilt, the use of nonselective β-blockers or β1-selective inhibitors was not associated with delayed neutrophil engraftment (Fig. 3G). The use of nonselective β-blockers, but not β1-selective inhibitors, was associated with delayed PLT engraftment (*B* = 11 days, *P* = 0.002 vs. *B* = 0.84, *P* = 0.74; Fig. 3H). We again observed known associations between delayed neutrophil engraftment and underlying myelodysplastic syndrome (34) and receipt of posttransplant cyclophosphamide (Fig. 3G; ref. 36), as well as delayed PLT engraftment and donor mismatch (Fig. 3H; ref. 34). RIC/NMA was also associated with delayed neutrophil engraftment (Fig. 3G). Aside from donor mismatch, none of these effects were as large as the effect of nonselective β-blocker use on PLT engraftment. These results mirror our observation in patients from UTSW, whose use of nonselective β-blockers was associated with substantially delayed PLT engraftment after allogeneic HCT.

To test if nonselective β-blockers impair hematopoietic regeneration in human BM, we evaluated the frequency of Lineage^−^CD34^+^CD38^−^ hematopoietic stem/progenitor cells in BM specimens obtained 100 days after patients received allogeneic HCT at UTSW on nonselective β-blockers versus age-matched controls not on a β-blocker (*n* = 5 and 4, mean age, 59 and 63 years, respectively). Patients taking nonselective β-blockers had a significantly lower frequency of Lineage^−^CD34^+^CD38^−^ cells (0.11%) than the controls (0.44%, *P* = 0.032: Supplementary Fig. S8A and S8B). This suggests that nonselective β-blockers may impair hematopoietic regeneration in human BM, as we observed in mice.

### Patients Transplanted with Fewer Cells Were Particularly Sensitive to β-Blockers

**Figure 4.**
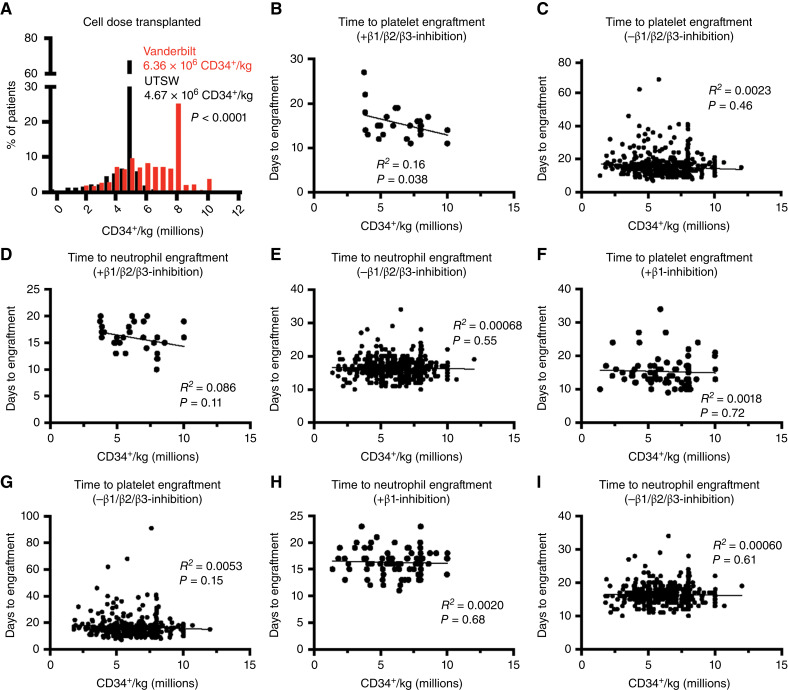
Nonselective β-blockers delay engraftment particularly when limiting numbers of cells are transplanted. **A,** Cell doses administered (CD34^+^ cells/kg) to patients receiving allogeneic HCT at UTSW and Vanderbilt. **B** and **C,** Correlation between cell dose administered and the time to PLT engraftment in patients who were (**B**), or were not (**C**), treated with nonselective β-blockers. **D** and **E,** Correlation between cell dose administered and the time to neutrophil engraftment in patients who were (**D**), or were not (**E**), treated with nonselective β-blockers. **F** and **G,** Correlation between cell dose administered and the time to PLT engraftment in patients who were treated with β1-selective inhibitors (**F**) vs. those not on any β-blocker (**G**). **H** and **I,** Correlation between cell dose administered and the time to neutrophil engraftment in patients administered β1-selective inhibitors (**H**) vs. those not on any β-blocker (**I**). Statistical significance was assessed using Student *t* tests (**A**) or bivariate analyses using Pearson correlation (**B–I**).

As we compared the data from UTSW and Vanderbilt, we found that patients who received allogeneic HCT at UTSW received fewer cells than those at Vanderbilt (mean of 4.7 × 10^6^ CD34^+^ cells/kg at UTSW vs. 6.4 × 10^6^ CD34^+^ cells/kg at Vanderbilt, *P* < 0.0001; Fig. 4A). We thus tested if patients transplanted with lower cell doses were more sensitive to the inhibitory effects of nonselective β-blockers. Patients at Vanderbilt received much more variable cell doses than those at UTSW (SD 1.9 × 10^6^ CD34^+^ cells/kg vs. 0.98 × 10^6^ CD34^+^ cells/kg; Fig. 4A), enabling an evaluation of the effect of nonselective β-blockers on the relationship between cell dose and neutrophil/PLT regeneration at Vanderbilt. In patients on nonselective β-blockers, there was a significant inverse correlation between cell dose and time to PLT engraftment (*R*^2^ = 0.16, *P* = 0.038; Fig. 4B), but not in patients who were not on a nonselective β-blocker (*R*^2^ = 0.0023, *P* = 0.46; Fig. 4C). Patients on nonselective β-blockers exhibited a nonsignificant trend toward an inverse correlation between cell dose and time to neutrophil engraftment (*R*^2^ = 0.086, *P* = 0.11; Fig. 4D), but not patients who were not on a nonselective β-blocker (*R*^2^ = 0.00068, *P* = 0.55; Fig. 4E). The use of β1-selective inhibitors had no significant effect on the correlation between cell dose and time to PLT or neutrophil engraftment (Fig. 4F–I). These results suggest that patients transplanted with lower cell doses may be particularly susceptible to the inhibitory effect of nonselective β-blockers on hematopoietic regeneration.

To test this in mice, we performed syngeneic transplants into carvedilol- or vehicle-treated C57BL/Ka mice using 3 × 10^5^, 6 × 10^5^, 12 × 10^5^, or 24 × 10^5^ C57BL/Ka BM cells. At lower doses of 3 × 10^5^ and 6 × 10^5^ BM cells, we generally observed significantly reduced blood cell counts, BM and spleen cellularity, and reduced frequencies of HSCs, MPPs, and LSK cells in the BM and spleen of carvedilol-treated as compared with vehicle-treated mice (Supplementary Fig. S9A–S9K). By contrast, when we transplanted 24 × 10^5^ BM cells, we observed no significant difference between carvedilol-treated and vehicle-treated mice (Supplementary Fig. S9A–S9K). In keeping with these observations, the carvedilol-treated mice exhibited significantly worse survival than vehicle-treated mice after receiving 3 × 10^5^ or 6 × 10^5^ BM cells but not at the 12 × 10^5^ or 24 × 10^5^ cell doses (Supplementary Fig. S9L). At the 24 × 10^5^ cell dose, the survival of carvedilol-treated and vehicle-treated mice was nearly indistinguishable (Supplementary Fig. S9L). The inhibitory effect of carvedilol on hematopoietic regeneration was thus overcome by transplanting higher doses of BM cells in mice.

**Figure 5.**
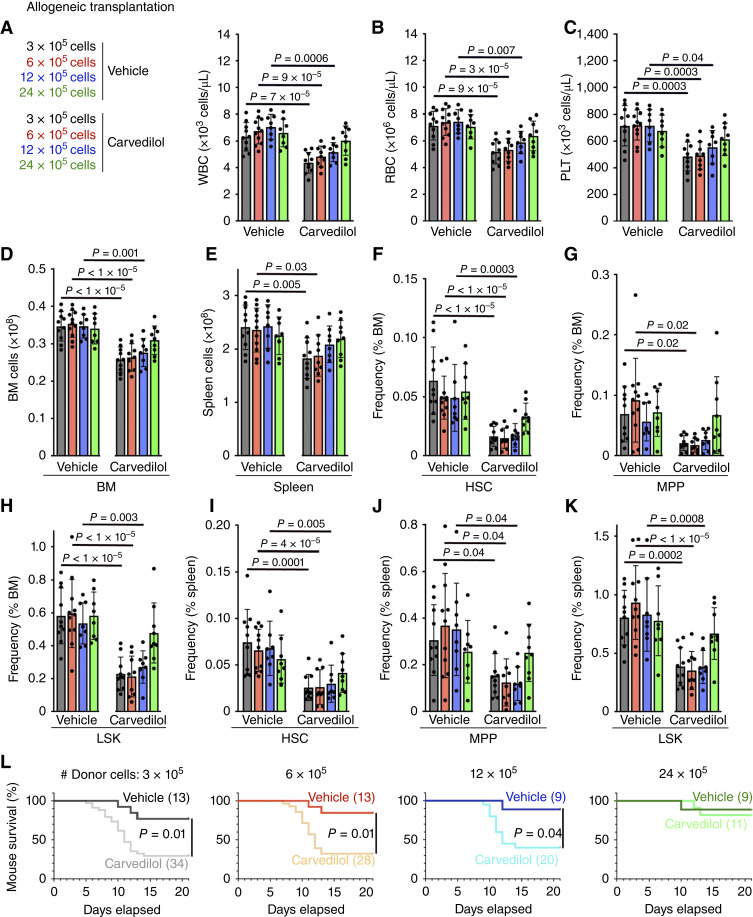
The inhibitory effect of carvedilol on hematopoietic regeneration after allogeneic transplantation can be overcome by transplanting larger doses of BM cells. Mice were treated with carvedilol or vehicle for 7 days before and 21 days after transplantation. We transplanted 3 × 10^5^, 6 × 10^5^, 12 × 10^5^, or 24 × 10^5^ T cell–depleted LP/J BM cells into irradiated C57BL/Ka recipients. **A–K,** A total of eight to 11 recipients per treatment from three independent experiments. Each dot represents a different mouse. All data represent mean ± SD. **A–C,** WBC (**A**), RBC (**B**), and PLT (**C**) counts from carvedilol-treated (right) or vehicle-treated (left) mice 21 days after transplantation. **D** and **E,** Total BM (**D**) and spleen (**E**) cellularity. **F–K,** The frequencies of HSCs, MPPs, and LSK cells in the BM (**F–H**) and spleen (**I–K**). **L,** Survival of carvedilol-treated and vehicle-treated mice after transplantation. The numbers of mice per treatment are shown in each panel. The statistical significance of differences among treatments was assessed using two-way ANOVAs followed by Sidak multiple comparison adjustments (**A–F**, **H**, **I**, and **K**), Mann–Whitney tests followed by Holm–Sidak multiple comparisons adjustments (**G** and **J**), or log-rank Mantel–Cox tests followed by Holm–Sidak multiple comparisons adjustment (**L**). All statistical tests were two sided.

We also tested this in the allogeneic context by transplanting 3 × 10^5^, 6 × 10^5^, 12 × 10^5^, or 24 × 10^5^ T cell–depleted LP/J BM cells into carvedilol- or vehicle-treated C57BL/Ka mice. As with the syngeneic transplants, at the 3 × 10^5^, 6 × 10^5^, and 12 × 10^5^ BM cell doses, we generally observed significantly reduced blood cell counts, BM and spleen cellularity, and reduced frequencies of HSCs, MPPs and LSK cells in the BM and spleen of carvedilol-treated as compared with vehicle-treated mice (Fig. 5A–K). At the 24 × 10^5^ cell dose, there was no significant difference between carvedilol- and vehicle-treated mice (Fig. 5A–K). The carvedilol-treated mice exhibited significantly worse survival than vehicle-treated mice after transplantation of 3 × 10^5^, 6 × 10^5^, or 12 × 10^5^ BM cells but not after transplanting 24 × 10^5^ cells (Fig. 5L). The inhibitory effect of carvedilol on hematopoietic regeneration in mice was thus overcome by transplanting higher doses of syngeneic or allogeneic BM cells.

### Patients Who Received Posttransplant Myelosuppressive Chemotherapy Were Particularly Sensitive to Nonselective β-Blockers

**Figure 6.**
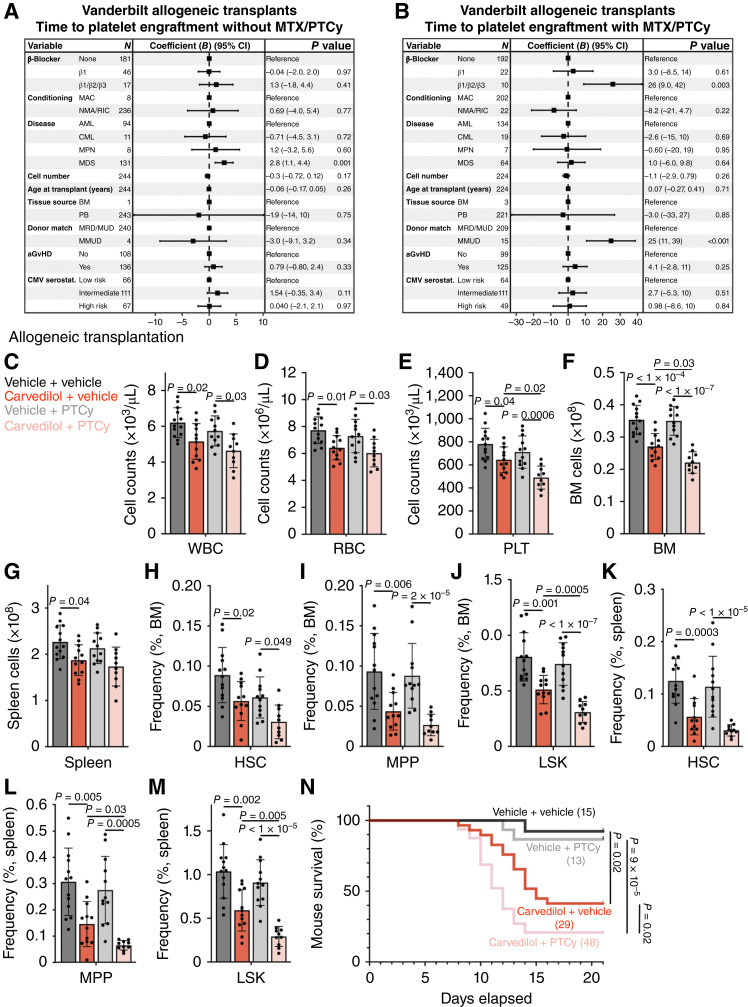
Posttransplant chemotherapy increased the inhibitory effect of nonselective β-blockers on engraftment after allogeneic transplantation in humans and mice. The risk factor coefficients (*B*) for time to PLT engraftment among patients at Vanderbilt who received allogeneic HCTs and who did not (**A**) or did (**B**) receive myelosuppressive GvHD prophylaxis with methotrexate or posttransplant cyclophosphamide. A generalized linear model was used, with covariates including age; β1-selective inhibitor use; nonselective β-blocker use; conditioning regimen [myeloablative conditioning (MAC) or RIC/NMA]; underlying disease [acute myeloid leukemia (AML), chronic myeloid leukemia (CML), myeloproliferative neoplasm (MPN), or myelodysplastic syndrome (MDS)]; cell dose (CD34^+^ cells/kg); cell source [BM or peripheral blood (PB)]; donor matching [matched related/matched unrelated donor (MRD/MUD), mismatched unrelated donor (MMUD), or haploidentical (Haplo)]; acute GvHD (aGvGD); and CMV serostatus (low risk = donor−/recipient−, intermediate risk = donor+/recipient+ or −, high risk = donor−/recipient+). *B* reflects the number of additional days required for neutrophil or PLT engraftment per unit of each predictive variable, with units being per year for age and binary (yes/no) for all other variables. *B* ± 95% confidence interval is shown. The dashed vertical line represents *B* of 0. **C**–**N,** Mice were treated with carvedilol or vehicle for 7 days before and 21 days after allogeneic transplantation of 6 × 10^5^ T cell–depleted LP/J BM cells into irradiated C57BL/Ka recipients. Cyclophosphamide (25 mg/kg body mass) or vehicle (PBS) were injected intraperitoneally at 3 and 4 days after transplantation. Each panel shows data from three independent experiments. **C**–**M,** A total of 10 to 13 recipients per treatment. Each dot represents a different mouse. All data represent mean ± SD. **C–E,** WBC (**C**), RBC (**D**), and PLT (**E**) counts at 21 days after transplantation. **F** and **G,** Total BM (**F**) and spleen (**G**) cellularity. **H–M,** The frequencies of HSCs, MPPs, and LSK cells in the BM (**H**–**J**) and spleen (**K**–**M**). **N,** Survival of carvedilol-treated and vehicle-treated mice with or without posttransplant cyclophosphamide treatment over time after transplantation. Data are from a total of 15 to 48 mice per condition in three independent experiments. The statistical significance of differences among treatments was assessed with one-way ANOVA followed by Sidak multiple comparisons adjustments (**C**–**K**), Welch’s one-way ANOVA followed by Dunnett T3 multiple comparison adjustments (**L** and **M**), or log-rank Mantel–Cox tests followed by Holm–Sidak multiple comparisons adjustment (**N**). All statistical tests were two sided.

Given that the use of nonselective β-blockers in humans was much more strongly associated with delayed engraftment in allogeneic as compared with autologous HCTs, we hypothesized that posttransplant myelosuppressive chemotherapy for prophylaxis of GvHD might increase dependence on β2/β3-adrenergic receptor signaling due to increased damage to hematopoietic niches. We were unable to evaluate this hypothesis in the UTSW allogeneic HCT cohort because nearly all of these patients received posttransplant myelosuppressive chemotherapy. However, at Vanderbilt, 219 recipients of allogeneic HCTs received posttransplant myelosuppressive chemotherapy and 244 did not. We compared the effects of β-blocker use in these two groups, performing a multivariate analysis using a generalized linear model to evaluate the effects of nonselective β-blockers and β1-selective inhibitors on engraftment, taking into account conditioning regimen, underlying disease, cell dose, age, cell source, donor mismatch, acute GvHD, and CMV serostatus. β-Blocker use did not significantly affect neutrophil engraftment in either group (Supplementary Fig. S10A and S10B). β1-selective inhibitors also did not significantly affect PLT engraftment in either group (Fig. 6A and B). Nonselective β-blocker use significantly delayed PLT engraftment in patients who received posttransplant chemotherapy (*B* = 26 days, *P* = 0.003, Fig. 6B) but not in patients who did not (*B* = 1.3, *P* = 0.41, Fig. 6A). The delayed PLT engraftment associated with nonselective β-blocker use in recipients of allogeneic HCT was thus strongly associated with exposure to posttransplant myelosuppressive chemotherapy.

To test whether posttransplant chemotherapy augments the effect of nonselective β-blockers on hematopoietic regeneration in mice, we injected cyclophosphamide on days 3 and 4 after allogeneic transplantation of 6 × 10^5^ T cell–depleted LP/J BM cells into C57BL/Ka mice that were treated with carvedilol or vehicle. We analyzed hematopoietic regeneration at 21 days after transplantation. Posttransplant cyclophosphamide itself did not significantly affect blood cell counts, BM or spleen cellularity, the frequencies of HSCs, MPPs, or LSK cells in the BM or spleen, or overall survival (OS; Fig. 6C–N). Carvedilol treatment in mice significantly reduced all of these parameters as compared with vehicle-treated mice (Fig. 6C–N). The combination of posttransplant cyclophosphamide with carvedilol significantly further reduced PLT counts (Fig. 6E), BM cellularity (Fig. 6F), LSK cell frequency in the BM (Fig. 6J) and MPP and LSK cell frequency in the spleen (Fig. 6L and M), as well as OS (Fig. 6N), as compared with carvedilol alone. Only 21% of mice that received both carvedilol and cyclophosphamide after transplantation survived, whereas 41% of mice that received carvedilol alone and 93% of control mice survived (Fig. 6N). These data suggest that additional damage to the niche after transplantation increases the dependence on β-adrenergic receptor signaling for hematopoietic regeneration.

### Use of Nonselective β-Blockers after HCT Is Associated with Worse Clinical Outcomes

**Figure 7.**
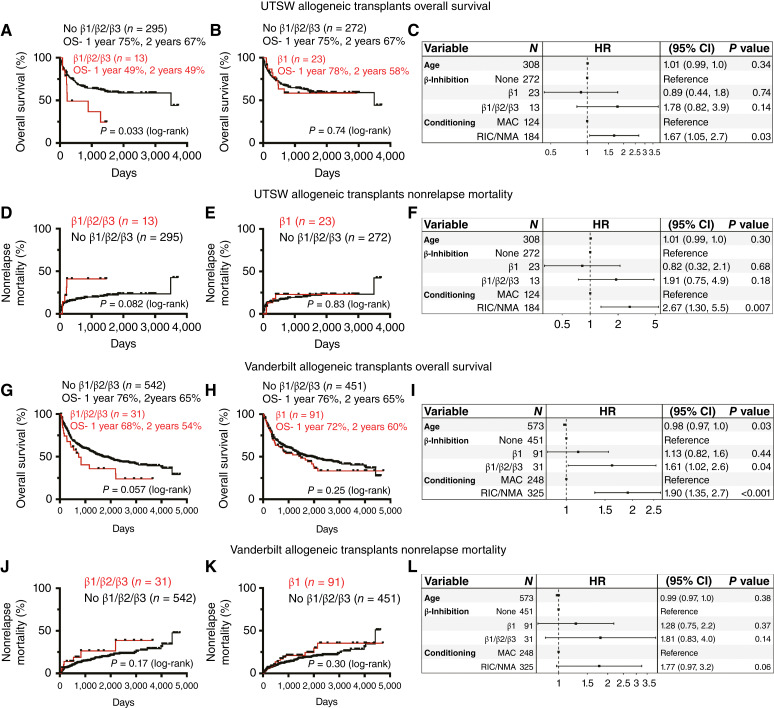
The use of nonselective β-blockers after transplantation is associated with worse clinical outcomes. **A** and **B,** Kaplan–Meier estimates of OS in patients receiving allogeneic HCT at UTSW who were, or were not, taking nonselective β-blockers (**A**) or β1-selective inhibitors vs. no β-blocker (**B**). One- and two-year OS (in years) is shown. **C,** The HR for OS in patients receiving allogeneic HCT at UTSW was estimated using a Cox proportional hazards model. **D** and **E,** Kaplan–Meier estimates of nonrelapse mortality (NRM) in patients receiving allogeneic HCT at UTSW who were, or were not, taking nonselective β-blockers (**D**) or β1-selective inhibitors vs. no β-blocker (**E**). **F,** The HR for NRM in patients receiving allogeneic HCT at UTSW was estimated using a Cox proportional hazards model. **G** and **H,** Kaplan–Meier estimates of OS in patients receiving allogeneic HCT at Vanderbilt who were, or were not, taking nonselective β-blockers (**G**) or β1-selective inhibitors vs. no β-blocker (**H**). **I,** The HR for OS in patients receiving allogeneic HCT at Vanderbilt was estimated as in **C**. **J** and **K,** Kaplan–Meier estimates of NRM in patients receiving allogeneic HCT at Vanderbilt who were, or were not, taking nonselective β-blockers (**J**) or β1-selective inhibitors vs. no β-blocker (**K**). **L,** The HR for NRM in patients receiving allogeneic HCT at Vanderbilt was estimated as in **F**. OS and NRM were compared between groups using a log-rank test (**A**, **B**, **D**, **E**, **G**, **H**, **J**, and **K**). For the Cox proportional hazards models (**C**, **F**, **I**, and **L**), covariates included age, conditioning regimen, nonselective β-blocker use, and β1-selective inhibitor use. The HR ± 95% confidence interval is shown, and the dashed vertical line represents a HR of 1.0.

We evaluated whether the use of nonselective β-blockers or β1-selective inhibitors was associated with differences in OS or nonrelapse mortality in patients who underwent allogeneic HCT at UTSW or Vanderbilt. Among patients who underwent allogeneic HCT at UTSW, the use of nonselective β-blockers was associated with significantly reduced OS as compared with patients not treated with nonselective β-blockers (median 7.6 vs. 116 months, *P* = 0.033; Fig. 7A): 1-year OS was 49% versus 75%, and 2-year OS was 49% versus 67%, respectively. Conversely, the use of β1-selective inhibitors did not significantly affect OS (median not reached in either group, *P* = 0.74; Fig. 7B): 1-year OS was 78% versus 75% and 2-year OS was 58% versus 67%, respectively.

We also evaluated the effect of β-blockers on OS in a multivariate analysis using a Cox proportional hazards model that took into account age and conditioning regimen as covariates. We found a nonsignificant trend toward nonselective β-blocker use, but not of β1-selective inhibitor, being associated with worse OS among patients with allogeneic HCT at UTSW (HR = 1.78, *P* = 0.14 vs. HR = 0.89, *P* = 0.74; Fig. 7C). Among the patients who received allogeneic HCT at UTSW, nonselective β-blocker use was also associated with a trend toward increased 5-year nonrelapse mortality (41.1% vs. 22.2%, *P* = 0.082; Fig. 7D), whereas β1-selective inhibitor use was not (22.9% vs. 21.9%, *P* = 0.83; Fig. 7E). All patients on nonselective β-blockers who exhibited nonrelapse mortality died between 82 and 228 days after transplantation, after neutrophil and PLT engraftment, suggesting delayed engraftment, rather than failure of engraftment, contributed to mortality. A multivariate analysis using the competing risks regression model (Fine–Gray), taking into account age and conditioning regimen, showed a nonsignificant trend toward nonselective β-blocker use being associated with worse nonrelapse mortality (HR = 1.91, *P* = 0.18; Fig. 7F). Thus, among the patients who received allogeneic HCT at UTSW, the use of nonselective β-blockers, but not β1-selective inhibitors, was associated with clinically significant trends toward worse OS and nonrelapse mortality.

Among patients who received allogeneic HCTs at Vanderbilt, the use of nonselective β-blockers was again associated with a borderline significant reduction in OS (median 25 vs. 56 months, *P* = 0.057; Fig. 7G): 1-year OS was 68% versus 76% and 2-year OS was 54% versus 65%. The use of β1-selective inhibitors did not significantly affect OS (median 49 vs. 64 months, *P* = 0.25; Fig. 7H): 1-year OS was 72% versus 76% and 2-year OS was 60% versus 65%. A multivariate analysis taking into account age and conditioning regimen demonstrated a significant association between nonselective β-blocker use, but not β1-selective inhibitor use, and reduced OS (HR = 1.6, *P* = 0.040 vs. HR = 1.1, *P* = 0.44; Fig. 7I). Among patients who recieved allogeneic HCT at Vanderbilt, no significant association was observed between β-blocker use and nonrelapse mortality (Fig. 7J–L). We thus observed clinically significant trends toward reduced survival among patients who received nonselective β-blockers, but not β1-selective inhibitors, after allogeneic HCT at UTSW and Vanderbilt.

To test if nonselective β-blocker use was associated with sequelae that might contribute to mortality after transplantation, we evaluated recipients who received allogeneic HCT for the incidences of infection and acute GvHD at any time after transplant, as well as the causes of death. Among recipients of allogeneic HCT at UTSW, we did not observe any statistically significant differences in the incidence of infection (Supplementary Fig. S11A and S11B) or acute GvHD (Supplementary Fig. S11C and S11D) in patients who received nonselective β-blockers or β1-selective inhibitors as compared with patients not treated with β-blockers. There was a significant increase in infections and organ failure, combined, as the cause of death among patients on nonselective β-blockers, but not β1-selective inhibitors, as compared with patients not treated with β-blockers (29% and 13% vs. 5.5%, *P* = 0.0029 and *P* = 0.32; Supplementary Fig. S11E and S11F). Among patients who received allogeneic HCT at Vanderbilt, we did not observe any statistically significant differences in the incidence of acute GvHD or specific causes of death in patients on nonselective β-blockers or β1-selective inhibitors as compared with patients not treated with β-blockers (Supplementary Fig. S12A–S12D).

### Discontinuation of β-Blockers Rescued Hematopoietic Regeneration

We tested in mice whether interruption of nonselective β-blocker administration around the time of transplantation would restore normal hematopoietic regeneration after transplantation. We treated mice with carvedilol or vehicle for 28 days prior to, and 21 days after, syngeneic BM transplantation, followed by an analysis of the blood cell counts and BM and spleen cellularity (Supplementary Fig. S13A). We also included a third treatment arm in which mice were treated with carvedilol from 28 to 7 days prior to transplantation, followed by vehicle treatment for the remainder of the experiment. The mice treated with carvedilol for the duration of the experiment had significantly reduced blood cell counts, BM cellularity, and frequencies of HSCs, MPPs, and LSK cells in the BM and spleen, as well as survival, when compared with mice treated with vehicle for the duration of the experiment or mice treated with vehicle beginning 7 days prior to transplantation (Supplementary Fig. S13B–S13M). Mice treated with vehicle for the duration of the experiment did not significantly differ from mice treated with vehicle beginning 7 days before transplantation in any hematopoietic parameter or in survival. These results suggest that discontinuation of nonselective β-blockers prior to transplantation is sufficient to preserve normal hematopoietic regeneration after transplantation.

## Discussion

By evaluating mice and independent patient cohorts from two institutions, we found that nonselective β-blockers impair hematopoietic regeneration after HCT. This effect was particularly pronounced in recipients of allogeneic HCT who received relatively low doses of CD34^+^ donor cells or posttransplant chemotherapy for GvHD prophylaxis, which is now widely used in allogeneic transplant recipients (36). This effect was not observed with β1-selective inhibitor use. These observations are consistent with the finding that β2- and β3-adrenergic receptor signaling are necessary in LepR^+^ BM stromal cells for hematopoietic regeneration after BM transplantation in mice (10). We found that the cessation of nonselective β-blockers in mice beginning 1 week before transplant and continuing for 3 weeks after transplant was sufficient to rescue hematopoietic regeneration after BMT (Supplementary Fig. S11). This suggests that a similar therapeutic strategy in patients could mitigate risk. An alternate strategy would be to switch patients on nonselective β-blockers to β1-selective inhibitors prior to HCT.

The administration of nonselective β-blockers significantly delayed engraftment after syngeneic transplants in mice and neutrophil engraftment (by 0.5 days) in autologous transplant recipients at UTSW but not at Vanderbilt. It is not clear why nonselective β-blockers significantly delayed engraftment after syngeneic transplants in mice but had little or no effect after autologous transplants in patients. Given that posttransplant chemotherapy was a major determinant of sensitivity to nonselective β-blockers among recipients of allogeneic transplant, it is possible that more myeloablative/niche-damaging conditioning regimens increase dependence on β-adrenergic receptor signaling and that whole-body irradiation in mice is more myeloablative, or more damaging to the niche, than the more optimized conditioning regimens used in humans for autologous transplants.

In patients, the most prominent effect of nonselective β-blocker use was a marked delay in PLT regeneration after allogeneic HCT. The etiology of persistent thrombocytopenia after allogeneic HCT is incompletely understood and likely multifactorial (41). Our study suggests that the use of nonselective β-blockers is a contributing factor. The approximately 2-week delay in PLT engraftment observed in both patients receiving allogeneic HCT at UTSW and Vanderbilt is highly clinically significant, as delayed PLT engraftment is associated with impaired OS and increased nonrelapse mortality after allogeneic HCT (29, 30). Such a delay in PLT engraftment would be associated with increased risks to patients from additional PLT transfusions, such as transfusion reactions, infections (42), and alloimmunization (43), as well as significant additional use of healthcare resources (44). Finally, the 3-to-4 day delay in neutrophil engraftment observed in patients receiving allogeneic HCT at UTSW would be associated with an equivalent increase in time to hospital discharge, which is comparable in magnitude to the effect of granulocyte colony-stimulating factor (G-CSF) administration on neutrophil engraftment (45).

Transplantation of lower CD34^+^ cell doses is associated with delayed engraftment (46), and we found that patients transplanted with low cell doses were particularly sensitive to the adverse effects of nonselective β-blockers. Thus, cessation of nonselective β-blockers may be particularly important in patients receiving low CD34^+^ cell doses, such as in the context of poor donor mobilization. Conversely, for patients in whom cessation of nonselective β-blockers is contraindicated, our studies in mice suggest that transplantation of higher cell doses may overcome the risk of delayed hematopoietic regeneration with continued nonselective β-blocker use. For such patients, special attention to donor mobilization, such as with the use of CXCR4 antagonists (47), should be considered. These results also raise the possibility that the use of donor HSC sources that tend to have limited CD34^+^ cell numbers, such as cord blood (48), may also confer increased sensitivity to the effects of nonselective β-blockers.

There are several limitations to this study. Due to the limited numbers of patients on β-blockers in each of the cohorts that we studied, our ability to compare the influences of nonselective β-blocker use in patients with different underlying diseases or prior therapies was limited. The vast majority of patients on β-blockers that we studied took carvedilol, and further studies in larger cohorts will be needed to determine whether there are differences among nonselective β-blockers in terms of their effects on hematopoietic regeneration. Although we show that transplantation of higher cell doses in mice can overcome the adverse effects of nonselective β-blockers, further studies are needed to determine the cell dose needed in humans to achieve similar effects. And although our studies show that transient discontinuation of nonselective β-blockers for 1 week before and 3 weeks after transplant can rescue hematopoietic regeneration in mice, further work is needed to test this in humans. Finally, our studies do not exclude an effect of nonselective β-blockers on other cell populations that can modulate engraftment such as NK or T cells (49, 50).

Future prospective clinical studies of nonselective β-blocker discontinuation around the time of transplantation are needed prior to widespread clinical application of our findings. It should also be tested whether the use of nonselective β-blockers affects hematopoietic regeneration in patients treated with chemotherapy outside of the context of HCT. Finally, future studies should test if β-agonists can accelerate hematopoietic regeneration after HCT.

In conclusion, our results identify the use of nonselective β-blockers as a risk factor for delayed hematopoietic regeneration after allogeneic HCT. Further exploration of the underlying mechanisms, and a refined understanding of the patients who are at the greatest risk, has the potential to identify tractable therapeutic strategies for improving clinical outcomes after HCT.

## Methods

### Mice

All mouse experiments complied with all relevant ethical regulations and were performed according to protocols approved by the Institutional Animal Care and Use Committees at the University of Texas Southwestern Medical Center (protocol 2019-102632-G). For syngeneic transplants, C57BL/Ka (CD45.2) BM cells were transplanted into irradiated C57BL/Ka-Thy-1.2 (CD45.1) recipients. For allogeneic transplants LP/J (Jackson strain # 000676), BM cells were transplanted into irradiated C57BL/Ka or C57BL/Ka-Thy-1.2 recipients. Mice were housed at the Animal Resource Center at the University of Texas Southwestern Medical Center in Association for Assessment and Accreditation of Laboratory Animal Care International–accredited, specific pathogen-free animal care facilities under a 12 hours:12 hours light:dark cycle with a temperature of 18°C to 24°C and humidity of 35% to 60%.

### Drug Administration to Mice

Carvedilol (APExBIO Technology, B1332) and metoprolol (APExBIO Technology, B1339) were reconstituted in DMSO, and stock solutions were kept frozen at −80°C until use. On the day of injection, the stock was diluted 1:20 with PBS and used immediately. 5% DMSO in PBS was used as vehicle control. Eight- to 12-week-old C57BL/Ka mice were treated daily with carvedilol (20 mg/kg body mass/day), metoprolol (20 mg/kg body mass/day), or vehicle control by intraperitoneal injection. For posttransplant cyclophosphamide treatment, cyclophosphamide (Amneal Pharmaceuticals, NDC 70121-1240-1, 25 mg/kg body mass) was intraperitoneally injected 3 and 4 days after BM transplantation as described in a prior study (51).

### Flow Cytometry

BM from one tibia and one femur was obtained by crushing the bones with a mortar and pestle and then dissociating into a single-cell suspension by gently triturating by pipette. Spleens were crushed between glass slides and then triturated. The cells were suspended in staining medium (Ca^2+^- and Mg^2+^-free Hank’s Balanced Salt Solution supplemented with 2% heat-inactivated bovine serum plus 1 mmol/L EDTA), filtered through a 40-μm nylon mesh, and counted by Vi-CELL XR (Beckman Coulter). For flow cytometric analysis, cells were stained with fluorophore-conjugated antibodies for 90 minutes on ice, including against the lineage markers CD2 (RM2-5, Tonbo, #35-0021), CD3 (17A2, BioLegend, #100204), CD5 (53-7.3, BioLegend, #100606), CD8a (53-6.7, Tonbo, #35-0081), Gr1 (RB6-8C5, BioLegend, #108406), Ter119 (TER-119, BioLegend, #116206), and B220 (RA3-6B2, BioLegend, #103206). Concurrently, we also stained using fluorophore-conjugated antibodies against c-Kit (2B8, Thermo Fisher Scientific, #47-1171-82), Sca-1 (D7, BioLegend, #122514), CD150 (TC15-12F12.2, BioLegend, #115904), and CD48 (HM48-1, BioLegend, #103426). All antibodies were used at 1:400 dilution. After staining, the cells were washed one time and resuspended in staining medium supplemented with propidium iodide at 1:1,000 dilution to exclude dead cells. Cells were analyzed using an FACSAria or FACSDiva (BD Biosciences) flow cytometer and FlowJo v9 software (Tree Star). The markers used to identify each cell population are shown in Supplementary Table S5; representative flow cytometry gates are shown in Supplementary Fig. S1W; and the key reagents and resources are listed in Supplementary Table S6.

### BM Transplantation

For syngeneic transplants, recipient mice [C57BL/Ka-Thy1.2 (CD45.1)] were irradiated using an XRad320 X-Ray irradiator (Precision X-Ray) with two doses of 540 rad at least 4 hours apart (1,080 rads total). Donor BM cells were obtained from C57BL/Ka (CD45.2) mice and injected into the retro-orbital venous sinus of the recipient mice. For allogeneic transplantation, C57BL/Ka (CD45.1 or CD45.2) mice were irradiated and transplanted with donor BM cells from LP/J mice. To avoid acute GvHD, T lymphocytes were depleted from LP/J BM cells before transplantation using paramagnetic beads (CD3ε MicroBead Kit, Miltenyi Biotec, #130-094-973). Briefly, the cells were stained with anti-CD3ε antibody conjugated to biotin (1:10 dilution) for 30 minutes on ice, then washed to eliminate unbound antibody, followed by the addition of anti-biotin microbeads (1:5 dilution) for 20 minutes on ice. The cells were washed one time to eliminate unbound microbeads, filtered through a 40-μm nylon mesh, and passed through the LS column (Miltenyi Biotec, #130-042-401) to eliminate T cells. The cells that were not retained in the column were collected, counted, and injected into the retro-orbital venous sinus of the recipient mice. Three weeks after transplantation, the mice were killed for analysis of hematopoietic regeneration (13, 52).

### IHC in Mouse BM Sections

Femurs were dissected from mice at 13 days after transplantation and fixed in 4% paraformaldehyde overnight, followed by 2 weeks of decalcification at 4°C in 10% EDTA plus 30% sucrose in PBS. Bones were sectioned and mounted on glass slides using the CryoJane Tape-Transfer System (Instrumedics). Slides were blocked with 5% bovine serum plus 0.2% Tween-20 in PBS for 1 hour at room temperature and incubated at 4°C overnight with anti-mouse CD41 antibody (MWReg30, BioLegend, #133901, 1:100) or anti-mouse Ly-6G (Gr1, clone RB6-8C5, Tonbo, #70-5931, 1:100). The next day, slides were washed three times for 10 minutes each with 0.2% Tween-20 in PBS and incubated at 4°C overnight with CF568 donkey anti-rat IgG (Biotium, #20092, 1:500) plus 4′,6-diamidino-2-phenylindole (1:1,000). The next day, slides were washed and mounted with ProLong Gold Antifade Mountant (Invitrogen). Images were acquired using Zeiss LSM780 or LSM880 confocal microscopes, and the number of CD41^+^ megakaryocytes or Gr1^+^ myeloid cells per field of view was counted from three randomly selected areas of bone.

### Clinical Cohorts and Data Collection

Clinical data were collected from all patients who underwent autologous and allogeneic HCT at UTSW between January 2013 and January 2023 (*n* = 871 and *n* = 311, respectively) and those who underwent allogeneic HCT at Vanderbilt between February 2010 and January 2023 (*n* = 549). Studies were conducted according to the principles embodied in the Declaration of Helsinki, and patient records were reviewed according to institutional review board (IRB)–approved protocols: UTSW STU-2020-0777 and Vanderbilt #230446. Both protocols have been determined by their respective IRBs to be exempt from informed consent, as no specific consent is needed for the statistical analysis of aggregated de-identified data. Data were collected on age, conditioning regimen (myeloablative conditioning vs. RIC/NMA), time to neutrophil and PLT engraftment (adjudicated as below), and nonselective or β1-selective β-blocker usage. Nonselective β-blockers included carvedilol, propranolol, and labetalol, and β1-selective β-blockers included metoprolol, atenolol, and bisoprolol. The IRB protocols provided an exemption from informed consent: no specific consent was needed for statistical analyses of aggregated de-identified data extracted from patient records. We complied with all relevant ethical regulations when performing this retrospective analysis.

### Clinical Engraftment and Survival Analyses

Time to neutrophil and PLT engraftment was adjudicated according to standard clinical criteria (33). Neutrophil engraftment was defined as the first of three consecutive days with an absolute neutrophil count ≥0.5 × 10^9^/L after transplant, and PLT engraftment was defined as the first of three consecutive days with a PLT count ≥20 × 10^9^/L (for patients from UTSW) or ≥50 × 10^9^/L (for patients from Vanderbilt) in the absence of PLT transfusion for seven consecutive days. The time to neutrophil and PLT engraftment was calculated from the day of transplantation. The time to engraftment was compared between propensity-matched groups using Student *t* tests. Single-variable and multivariate linear regression analyses were conducted using a generalized linear model to evaluate the effects of β-blocker use on the time to engraftment, considering other clinical variables such as age, CD34^+^ cell dose, and conditioning regimen. Kaplan–Meier survival curves were used to assess differences in OS and nonrelapse mortality. A multivariate Cox proportional hazards model was used to evaluate the impact of β-blockers on OS and considering the effects of age and conditioning regimens. A competing risks regression model [Fine–Gray (53)] was used to evaluate the impact of β-blockers on nonrelapse mortality and accounting for relapse as a competing event and considering the effects of age and the conditioning regimen. All analyses were performed using R (version 4.3.3).

### Accrual and Analysis of Patient Specimens

Human BM specimens were obtained from the Hematologic Malignancies tissue bank at UTSW with IRB approval (UTSW STU-2019-0815) and the written informed consent from patients. These studies were conducted according to the principles embodied in the Declaration of Helsinki. The hematopoietic stem and progenitor cell frequency was assessed using antibodies against CD34 (581) from BioLegend and CD38 (HIT2) from eBioscience. We additionally used a lineage cocktail containing antibodies against CD2 (RPA-2.10), CD3 (HIT3a), CD4 (RPA-T4), CD7 (M-T701), CD8 (RPA-T8), CD10 (HI10a), CD11b (ICRF44), CD14 (TuK4), CD19 (CC2C6), CD20 (2H7), GPA (HIR2), and CD56 (B159), all from BD Biosciences. Cells were analyzed using a FACSAria flow cytometer and FlowJo v10 software (Tree Star). The markers used to identify Lineage-CD34^+^CD38^−^ cells are shown in Supplementary Table S5 and the representative flow cytometry gates are shown in Supplementary Fig. S8A.

### Statistics and Reproducibility

The number of mice analyzed and the number of independent experiments are indicated in each figure legend. Mice were allocated to experiments randomly and samples processed in an arbitrary order, but formal randomization techniques were not used. Data collection and analysis were not blinded to experimental treatments. Samples sizes were not predetermined based on statistical power calculations but were similar to those used in prior publications (54).

Prior to analyzing the statistical significance of differences among treatments, we tested whether data were normally distributed and whether variance was similar among groups. To test for normality, we performed the Shapiro–Wilk tests when 3 ≤ *n* < 20 or D’Agostino omnibus tests when *n* ≥ 20. To test whether variability significantly differed among groups, we performed *F*-tests (for experiments with two groups) or Levene median tests (for experiments with more than two groups). When the data significantly deviated from normality or variability significantly differed among groups, we log_2_-transformed the data and tested again for normality and variability. If the transformed data no longer significantly deviated from normality and equal variability, we performed parametric tests on the transformed data. If log_2_-transformation was not possible or the transformed data still significantly deviated from normality or equal variability, we performed nonparametric tests on the nontransformed data.

When data or log_2_-transformed data were normal and equally variable, statistical analyses were performed using Student *t* tests (when there were two groups), one-way ANOVA (when there were more than two groups), or two-way ANOVA/matched samples two-way ANOVA (when there were two or more groups with multiple cell populations, tissues, or cell doses). When the data or log_2_-transformed data were normally distributed but unequally variable, statistical analysis was performed using Welch’s *t* tests (when there were two groups) or Welch’s one-way ANOVA (when there were more than two groups). When the data or log_2_-transformed data were abnormally distributed, statistical analysis was performed using Mann–Whitney tests (when there were two groups). After one-way ANOVA, *P* values from multiple comparisons were adjusted using Tukey method (when all the pairwise comparisons were performed) or Sidak method (when planned comparisons were performed). After Welch’s one-way ANOVA, *P* values from multiple comparisons were adjusted using Dunnett T3 method. After two-way ANOVA/matched samples two-way ANOVAs, *P* values from multiple comparisons were adjusted using Sidak method (when there were more than two groups and planned comparisons). Log-rank tests were used to assess the statistical significance of survival differences. The Holm–Sidak method was used to adjust comparisons involving multiple Student *t* tests, Welch’s *t* tests, Mann–Whitney tests, or log-rank tests. All statistical tests were two-sided. All data represent mean ± SD. Statistical tests were performed using GraphPad Prism V10.2.3. No custom computer code was used in this study.

### Data Availability

This study did not involve datasets that required submission to public repositories. There are no restrictions on the availability of de-identified data. All other data supporting the findings of this study are available from the corresponding authors on reasonable request.

## Supplementary Material

Supplementary Table 1 Supplementary Table S1. Characteristics of UTSW autologous transplant patients. Continuous measures are shown as mean (SD) and categorical measures as percentages. A one-way ANOVA was used to compare continuous variables, and a χ^2^ test was used to compare categorical measures.

Supplementary Table 2 Supplementary Table S2. Characteristics of UTSW allogeneic transplant patients. Abbreviations: Acute myeloid leukemia (AML), acute lymphoblastic leukemia (ALL), myelodysplastic syndrome (MDS), myeloproliferative neoplasm (MPN), mixed-phenotype acute leukemia (MPAL), severe aplastic anemia (sAA), myeloablative conditioning (MAC), nonmyeloablative conditioning (NMA), reduced-intensity conditioning (RIC), matched related donor (MRD), matched unrelated donor (MUD), mismatched unrelated donor (MMUD), haploidentical (Haplo), graft-versus-host disease (GvHD), post-transplant cytoxan (PTCy), methotrexate (MTX), and cytomegalovirus (CMV). Continuous measures are shown as mean (SD), and categorical measures as percentages. A one-way ANOVA was used to compare continuous variables and a χ^2^ test was used to compare categorical measures.

Supplementary Table 3 Supplementary Table S3. Characteristics of Vanderbilt autologous transplant patients. Continuous measures are shown as mean (SD), and categorical measures as percentages. A one-way ANOVA was used to compare continuous variables, and a χ^2^ test was used to compare categorical measures.

Supplementary Table 4 Supplementary Table S4. Characteristics of Vanderbilt allogeneic transplant patients. Abbreviations: Acute myeloid leukemia (AML), myelodysplastic syndrome (MDS), myeloproliferative neoplasm (MPN), mixed-phenotype acute leukemia (MPAL), myeloablative conditioning (MAC), non-myeloablative conditioning (NMA), reduced-intensity conditioning (RIC), matched related donor (MRD), matched unrelated donor (MUD), mismatched unrelated donor (MMUD), haploidentical (Haplo), graft-versus-host disease (GvHD), post-transplant cytoxan (PTCy), methotrexate (MTX), and cytomegalovirus (CMV). Continuous measures are shown as Mean (SD), and categorical measures as proportions. A one-way ANOVA test was used to compare continuous variables, and a χ^2^ test was used to compare categorical measures.

Supplementary Table 5 Supplementary Table S5. Related to Fig. 1-4 and Supplementary Fig. S3. Cell populations analyzed by flow cytometry in this study.

Supplementary Table 6 Supplementary Table S6. Related to Fig. 1-7. Key Resources Table.

Supplementary Figure 1 Supplementary Figure S1. Carvedilol and metoprolol do not affect steady-state hematopoiesis.

Supplementary Figure 2 Supplementary Figure S2: Metoprolol treatment did not significantly affect hematopoietic regeneration after syngeneic or allogeneic transplantation in mice.

Supplementary Figure 3 Supplementary Figure S3: Propensity matched analysis of the effect of non-selective b adrenergic receptor inhibitors on hematopoietic regeneration in Vanderbilt patients undergoing autologous and allogeneic transplantation.

Supplementary Figure 4 Supplementary Figure S4: Clinical variables associated with time to hematopoietic regeneration after autologous transplantation at UTSW.

Supplementary Figure 5 Supplementary Figure S5: Clinical variables associated with time to hematopoietic regeneration after allogeneic transplantation at UTSW.

Supplementary Figure 6 Supplementary Figure S6: Clinical variables associated with time to hematopoietic regeneration after autologous transplantation in Vanderbilt patients.

Supplementary Figure 7 Supplementary Figure S7: Clinical variables associated with time to hematopoietic regeneration after allogeneic transplantation at Vanderbilt.

Supplementary Figure 8 Supplementary Figure S8: Hematopoietic stem and progenitor cell frequencies after allogeneic transplantation.

Supplementary Figure 9 Supplementary Figure S9: The inhibitory effect of carvedilol on hematopoietic regeneration after syngeneic transplantation can be overcome by transplanting larger doses of bone marrow cells.

Supplementary Figure 10 Supplementary Figure S10: Post-transplant chemotherapy or use of non-selective b blockers was not associated with changes in neutrophil engraftment after allogeneic transplantation in Vanderbilt allogeneic HCT patients.

Supplementary Figure 11 Supplementary Figure S11: Infection, graft-versus-host disease, and causes of death in UTSW allogeneic HCT recipients.

Supplementary Figure 12 Supplementary Figure S12: Infection, graft-versus-host disease, and causes of death in Vanderbilt allogeneic HCT recipients.

Supplementary Figure 13 Supplementary Figure S13: Discontinuation of b blockers around the time of transplantation rescues hematopoietic regeneration.
