# Supplementary material for: Nonselective β-Adrenergic Receptor Inhibitors Impair Hematopoietic Regeneration in Mice and Humans after Hematopoietic Cell Transplants
Source: Cancer Discov. 2024 Dec 30;15(4):748–66. doi: 10.1158/2159-8290.CD-24-0719 (PMC11962394; doi:10.1158/2159-8290.CD-24-0719)
Supplement: Supplementary Table 1 — Supplementary Table S1. Characteristics of UTSW autologous transplant patients. Continuous measures are shown as mean (SD) and categorical measures as percentages. A one-way ANOVA was used to compare continuous variables, and a χ2 test was used to compare categorical measures. [file cd-24-0719_supplementary_table_1_suppst1.pdf]

Supplementary Table S1. Characteristics of UTSW autologous transplant patients. Continuous measures are shown as mean (SD) and categorical measures as percentages. A one-way ANOVA was used to compare continuous variables, and a Chi-squared test was used to compare categorical measures.

**Characteristic**	No β-blocker use (n = 675)	Non-selective β-blocker use (n = 73)	β1-selective inhibitor use (n = 123)	
**Age** (SD)	57.3 (12.6)	62.0 (9.48)	59.1 (11.2)	*P*=0.0038
**Sex**				*P*=0.153
Male, n (%)	388 (57.5)	50 (60.8)	76 (61.8)	
Female, n (%)	287 (42.5)	23 (31.5)	47 (38.2)	
**Race**				*P*=0.0002
Black, n (%)	116 (17.2)	14 (19.2)	39 (31.7)	
White, n (%)	441 (65.3)	48 (65.8)	74 (60.2)	
Hispanic, n (%)	89 (13.2)	7 (9.59)	6 (4.88)	
Asian, n (%)	26 (3.85)	1 (1.37)	3 (2.44)	
Other, n (%)	3 (0.444)	3 (4.11)	1 (0.813)	
**Underlying disease**				*P*=0.209
Non-hodgkin's lymphoma, n (%)	145 (21.5)	11 (15.1)	18 (14.6)	
Hodgkin's lymphoma, n (%)	33 (4.89)	2 (2.74)	5 (4.07)	
Plasma cell dyscrasia, n (%)	464 (68.7)	60 (82.2)	95 (77.2)	
Germ cell tumor, n (%)	29 (4.30)	0 (0)	5 (4.07)	
Other, n (%)	4 (0.593)	0 (0)	0 (0)	
