# Supplementary material for: Nonselective β-Adrenergic Receptor Inhibitors Impair Hematopoietic Regeneration in Mice and Humans after Hematopoietic Cell Transplants
Source: Cancer Discov. 2024 Dec 30;15(4):748–66. doi: 10.1158/2159-8290.CD-24-0719 (PMC11962394; doi:10.1158/2159-8290.CD-24-0719)
Supplement: Supplementary Table 2 — Supplementary Table S2. Characteristics of UTSW allogeneic transplant patients. Abbreviations: Acute myeloid leukemia (AML), acute lymphoblastic leukemia (ALL), myelodysplastic syndrome (MDS), myeloproliferative neoplasm (MPN), mixed-phenotype acute leukemia (MPAL), severe aplastic anemia (sAA), myeloablative conditioning (MAC), nonmyeloablative conditioning (NMA), reduced-intensity conditioning (RIC), matched related donor (MRD), matched unrelated donor (MUD), mismatched unrelated donor (MMUD), haploidentical (Haplo), graft-versus-host disease (GvHD), post-transplant cytoxan (PTCy), methotrexate (MTX), and cytomegalovirus (CMV). Continuous measures are shown as mean (SD), and categorical measures as percentages. A one-way ANOVA was used to compare continuous variables and a χ2 test was used to compare categorical measures. [file cd-24-0719_supplementary_table_2_suppst2.pdf]

Supplementary Table S2. Characteristics of UTSW allogeneic transplant patients.

Abbreviations: Acute myeloid leukemia (AML), acute lymphoblastic leukemia (ALL), myelodysplastic syndrome (MDS), myeloproliferative neoplasm (MPN), mixed-phenotype acute leukemia (MPAL), severe aplastic anemia (sAA), myeloablative conditioning (MAC), non-myeloablative conditioning (NMA), reduced-intensity conditioning (RIC), matched related donor (MRD), matched unrelated donor (MUD), mismatched unrelated donor (MMUD), haploidentical (Haplo), graft-versus-host disease (GvHD), post-transplant cytoxan (PTCy), methotrexate (MTX), and cytomegalovirus (CMV). Continuous measures are shown as mean (SD), and categorical measures as percentages. A one-way ANOVA was used to compare continuous variables and a Chi-squared test was used to compare categorical measures.

**Characteristic**	No β-blocker use (n = 272)	Non-selective β-blocker use (n = 14)	β1-selective inhibitor use (n = 23)	
**Age** (SD)	49.7 (14.3)	56.5 (17.2)	60.4 (7.43)	*P*=0.0008
**Sex**				*P*=0.206
Male, n (%)	149 (54.8)	8 (57.1)	17 (73.9)	
Female, n (%)	123 (45.2)	6 (42.9)	6 (26.1)	
**Race**				*P*=0.64
Black, n (%)	25 (9.19)	2 (14.3)	4 (17.4)	
White, n (%)	196 (72.1)	11 (78.6)	16 (69.6)	
Hispanic, n (%)	36 (13.2)	1 (7.14)	2 (8.70)	
Asian, n (%)	12 (4.41)	0 (0)	0 (0)	
Other, n (%)	3 (1.10)	0 (0)	1 (4.35)	
**Underlying disease**				*P*=0.0979
AML, n (%)	115 (42.3)	7 (50.0)	6 (26.1)	

ALL, n (%)	51 (18.8)	1 (14.3)	1 (4.35)	
MDS, n (%)	49 (18.0)	3 (21.4)	10 (43.5)	
CML, n (%)	5 (1.84)	0 (0)	1 (4.35)	
MPAL, n (%)	4 (1.47)	0 (0)	0 (0)	
MPN, n (%)	4 (1.47)	1 (14.3)	1 (4.35)	
Lymphoma, n (%)	30 (11.0)	0 (0)	4 (17.4)	
sAA, n (%)	10 (3.68)	0 (0)	0 (0)	
Other, n (%)	4 (1.47)	1 (14.3)	0 (0)	
**Conditioning**				
MAC, n (%)	117 (43.0)	3 (28.6)	4 (17.4)	*P*=0.0190
NMA/RIC, n (%)	155 (57.0)	11 (78.6)	19 (82.6)	
**Cell number** (x10^{6} 34+/kg) (SD)	5.07 (5.79)	4.51 (0.887)	4.96 (0.307)	*P*=0.939
**Cell source**				*P*=0.166
Bone marrow	37 (13.6)	2 (14.3)	0 (0)	
Peripheral blood	235 (86.4)	12 (85.7)	23 (100)	
**Donor match**				*P*=0.0598
MRD/MUD	213 (78.3)	8 (57.1)	14 (60.9)	
MMUD	16 (5.88)	1 (7.14)	4 (17.4)	
Haplo	43 (15.8)	5 (35.7)	5 (21.7)	
**Myelosuppressive GvHD prophylaxis**				*P*<0.0001
None, n (%)	6 (2.21)	0 (0)	7 (30.4)	
PTCy, n (%)	167 (61.4)	12 (85.7)	13 (56.5)	
MTX, n (%)	99 (36.4)	2 (14.3)	3 (13.0)	
**Acute GvHD**	162 (59.6)	8 (57.1)	15 (65.2)	*P*=0.849

**Chronic GvHD**	113 (41.5)	2 (14.3)	12 (52.2)	*P*=0.0691
**CMV serostatus**				*P*=0.662
low risk, n (%)	62 (22.8)	5 (35.7)	6 (26.1)	
intermediate risk, n (%)	151 (55.5)	8 (57.1)	12 (52.2)	
high risk, n (%)	59 (21.7)	1 (7.14)	5 (21.7)	
