# Supplementary material for: Nonselective β-Adrenergic Receptor Inhibitors Impair Hematopoietic Regeneration in Mice and Humans after Hematopoietic Cell Transplants
Source: Cancer Discov. 2024 Dec 30;15(4):748–66. doi: 10.1158/2159-8290.CD-24-0719 (PMC11962394; doi:10.1158/2159-8290.CD-24-0719)
Supplement: Supplementary Table 3 — Supplementary Table S3. Characteristics of Vanderbilt autologous transplant patients. Continuous measures are shown as mean (SD), and categorical measures as percentages. A one-way ANOVA was used to compare continuous variables, and a χ2 test was used to compare categorical measures. [file cd-24-0719_supplementary_table_3_suppst3.pdf]

Supplementary Table S3. Characteristics of Vanderbilt autologous transplant patients. Continuous measures are shown as mean (SD), and categorical measures as percentages. A one-way ANOVA was used to compare continuous variables, and a Chi-squared test was used to compare categorical measures.

**Characteristic**	No β-blocker use (n = 833)	Non-selective β-blocker use (n = 74)	β1-selective inhibitor use (n = 213)	
**Age** (SD)	57.3 (11.4)	59.7 (9.35)	62.3 (9.39)	*P*<0.0001
**Underlying disease**				*P*<0.0001
Non-hodgkin's lymphoma, n (%)	132 (15.8)	10 (13.5)	29 (13.6)	
Hodgkin's lymphoma, n (%)	52 (6.24)	2 (2.70)	4 (1.88)	
Plasma cell dyscrasia, n (%)	627 (75.3)	62 (83.8)	173 (81.2)	
Germ cell tumor, n (%)	20 (2.40)	0 (0)	7 (3.29)	
Other, n (%)	2 (0.240)	0 (0)	0 (0)	
