# Supplementary material for: Nonselective β-Adrenergic Receptor Inhibitors Impair Hematopoietic Regeneration in Mice and Humans after Hematopoietic Cell Transplants
Source: Cancer Discov. 2024 Dec 30;15(4):748–66. doi: 10.1158/2159-8290.CD-24-0719 (PMC11962394; doi:10.1158/2159-8290.CD-24-0719)
Supplement: Supplementary Table 4 — Supplementary Table S4. Characteristics of Vanderbilt allogeneic transplant patients. Abbreviations: Acute myeloid leukemia (AML), myelodysplastic syndrome (MDS), myeloproliferative neoplasm (MPN), mixed-phenotype acute leukemia (MPAL), myeloablative conditioning (MAC), non-myeloablative conditioning (NMA), reduced-intensity conditioning (RIC), matched related donor (MRD), matched unrelated donor (MUD), mismatched unrelated donor (MMUD), haploidentical (Haplo), graft-versus-host disease (GvHD), post-transplant cytoxan (PTCy), methotrexate (MTX), and cytomegalovirus (CMV). Continuous measures are shown as Mean (SD), and categorical measures as proportions. A one-way ANOVA test was used to compare continuous variables, and a χ2 test was used to compare categorical measures. [file cd-24-0719_supplementary_table_4_suppst4.pdf]

Supplementary Table S4. Characteristics of Vanderbilt allogeneic transplant patients. Abbreviations: Acute myeloid leukemia (AML), myelodysplastic syndrome (MDS), myeloproliferative neoplasm (MPN), mixed-phenotype acute leukemia (MPAL), myeloablative conditioning (MAC), non-myeloablative conditioning (NMA), reduced-intensity conditioning (RIC), matched related donor (MRD), matched unrelated donor (MUD), mismatched unrelated donor (MMUD), haploidentical (Haplo), graft-versus-host disease (GvHD), post-transplant cytoxan (PTCy), methotrexate (MTS), and cytomegalovirus (CMV). Continuous measures are shown as Mean (SD), and categorical measures as proportions. A one-way ANOVA test was used to compare continuous variables, and a Chi-squared test was used to compare categorical measures.

**Characteristic**	No β-blocker use (n = 453)	Non-selective β-blocker use (n = 33)	β1-selective inhibitor use (n = 93)	
**Age** (SD)	53.3 (13.1)	57.0 (9.50)	58.5 (10.7)	*P*=0.0007
**Underlying disease**				*P*=0.922
AML, n (%)	225 (49.7)	20 (60.6)	42 (45.2)	
MDS, n (%)	183 (40.4)	10 (30.3)	41 (44.1)	
CML, n (%)	29 (6.40)	2 (6.06)	5 (5.38)	
MPAL, n (%)	2 (0.442)	0 (0)	1 (1.08)	
MPN, n (%)	12 (2.65)	1 (3.03)	4 (4.30)	
Other, n (%)	2 (0.442)	0 (0)	0 (0)	
**Conditioning**				
MAC, n (%)	214 (47.2)	11 (33.3)	26 (28.0)	*P*=0.0014
NMA/RIC, n (%)	239 (52.8)	22 (66.7)	67 (72.0)	

**Cell number** ($x10^6$ 34+/kg) (SD)	6.39 (1.84)	6.31 (1.84)	6.22 (2.08)	*P*=0.733
**Cell source**				*P*=0.319
Bone marrow	21 (4.64)	0 (0)	6 (6.45)	
Peripheral blood	432 (95.4)	33 (100)	87 (93.5)	
**Donor match**				*P*=0.0689
MRD/MUD	435 (96.0)	29 (87.9)	90 (96.8)	
MMUD/Haplo	18 (3.97)	4 (12.1)	3 (3.23)	
**Myelosuppressive GvHD prophylaxis**				*P*=0.0321
None, n (%)	231 (51.0)	21 (63.6)	63 (67.7)	
PTCy, n (%)	11 (2.43)	0 (0)	1 (1.08)	
MTX, n (%)	211 (46.6)	12 (36.4)	29 (31.2)	
**Acute GvHD**	238 (52.5)	17 (51.5)	52 (55.9)	*P*=0.825
**Chronic GvHD**	200 (44.2)	12 (36.4)	46 (49.5)	*P*=0.400
**CMV serostatus**				*P*=0.608
low risk, n (%)	127 (28.0)	9 (27.2)	19 (20.4)	
intermediate risk, n (%)	276 (60.9)	21 (63.6)	61 (65.6)	
high risk, n (%)	50 (11.0)	3 (9.09)	13 (14.0)	
