# Supplementary material for: Nonselective β-Adrenergic Receptor Inhibitors Impair Hematopoietic Regeneration in Mice and Humans after Hematopoietic Cell Transplants
Source: Cancer Discov. 2024 Dec 30;15(4):748–66. doi: 10.1158/2159-8290.CD-24-0719 (PMC11962394; doi:10.1158/2159-8290.CD-24-0719)
Supplement: Supplementary Table 5 — Supplementary Table S5. Related to Fig. 1-4 and Supplementary Fig. S3. Cell populations analyzed by flow cytometry in this study. [file cd-24-0719_supplementary_table_5_suppst5.pdf]

Supplementary Table S5. Related to Fig. 1-4 and Supplementary Fig. S3. Cell populations analyzed by flow cytometry in this study.

Cell population	Abbreviation	Markers	Reference
**Mouse**			
Hematopoietic Stem Cells	HSC	$CD150^{+}CD48^{-}Lineage^{-}Sca1^{+}c\text{-}kit^{+}$	(1)
Multipotent Progenitors	MPP	$CD150^{-}CD48^{-}Lineage^{-}Sca1^{+}c\text{-}kit^{+}$	(1)
$Lineage^{-}$-$Sca1^{+}$-$c\text{-}Kit^{+}$ hematopoietic progenitors	LSK	$Lineage^{-}Sca1^{+}c\text{-}kit^{+}$	
**Human**			
Hematopoietic stem and progenitor cells	HSPC	$Lineage^{-}CD34^{+}CD38^{-}$	(2)

REFERENCES

1. Kiel MJ, Yilmaz OH, Iwashita T, Yilmaz OH, Terhorst C, Morrison SJ. SLAM family receptors distinguish hematopoietic stem and progenitor cells and reveal endothelial niches for stem cells. Cell **2005**;121(7):1109-21 doi 10.1016/j.cell.2005.05.026.

2. Majeti R, Park CY, Weissman IL. Identification of a hierarchy of multipotent hematopoietic progenitors in human cord blood. Cell Stem Cell **2007**;1(6):635-45 doi S1934-5909(07)00218-4 [pii]
10.1016/j.stem.2007.10.001.
