# Supplementary material for: Nonselective β-Adrenergic Receptor Inhibitors Impair Hematopoietic Regeneration in Mice and Humans after Hematopoietic Cell Transplants
Source: Cancer Discov. 2024 Dec 30;15(4):748–66. doi: 10.1158/2159-8290.CD-24-0719 (PMC11962394; doi:10.1158/2159-8290.CD-24-0719)
Supplement: Supplementary Table 6 — Supplementary Table S6. Related to Fig. 1-7. Key Resources Table. [file cd-24-0719_supplementary_table_6_suppst6.pdf]

Supplementary Table S6. Related to Fig. 1-7. Key Resources Table.

REAGENT or RESOURCE	SOURCE	IDENTIFIER
**Experimental models: Organisms/strains**		
C57BL/Ka-Thy-1.2 (CD45.1)	Irving Weissman lab	
LP/J	Jackson laboratory	RRID:IMSR_JAX:000676
C57BL/Ka (CD45.2)	Irving Weissman lab	

**Chemicals**		
Carvedilol	APExBIO	Cat# B1332
Metoprolol Tartrate	APExBIO	Cat# B1339
Cyclophosmphamide	Amneal Pharmaceuticals	NDC 70121-1240-1

**Antibodies**		
FITC Anti-Mouse CD2 (RM2-5)	Tonbo Biosciences	Cat# 35-0021; RRID:AB_2621657
FITC Anti-Mouse CD3 (17A2)	Biolegend	Cat# 100204; RRID:AB_312661
FITC Anti-Mouse CD5 (53-7.3)	Biolegend	Cat# 100606; RRID:AB_312735
FITC Anti-Mouse CD8a (53-6.7)	Tonbo Biosciences	Cat# 35-0081; RRID:AB_2621671
FITC Anti-Mouse Gr1 (RB6-8C5)	Biolegend	Cat# 108406; RRID:AB_313371
FITC Anti-Mouse Ter119 (TER-119)	Tonbo Biosciences	Cat# 35-5921; RRID:AB_2621720
FITC Anti-Mouse B220 (RA3-6B2)	Biolegend	Cat# 103206; RRID:AB_312991
CD117 (c-kit) mAb (2B8), APC-eFluor780	Thermo Fisher Scientific	Cat# 47-1171-82; RRID:AB_1272177
PE/Cy7 anti mouse Ly-6A/E (Sca-1) (D7)	Biolegend	Cat# 122514; RRID:AB_756199
PE anti-mouse CD150 (SLAM)	Biolegend	Cat# 115904; RRID:AB_313683
Alexa Fluor(R) 700 anti-mouse CD48 (HM48-1)	Biolegend	Cat# 103426; RRID:AB_10612755
CD3ε-Biotin, mouse	Myltenyi Biotec.	Cat# 130-101-878; RRID:AB_2657926
Anti-Biotin MicroBeads	Myltenyi Biotec.	Cat# 130-090-485; RRID:AB_244365
Anti-mouse CD41 mAb (MWReg30)	BioLegend	Cat #133901; RRID:AB_1626143
Anti-Mouse Ly-6G mAb (Gr-1) (RB6-8C5)	Tonbo Biosciences	Cat# 70-5931; RRID:AB_2621525
Donkey Anti-Rat IgG (H+L), CF568	Biotium	Cat# 20092; RRID:AB_10559037
Pacific Blue (TM) anti-human CD34 (581)	Biolegend	Cat# 343511; RRID:AB_1877198
CD38 Monoclonal Antibody (HIT2), PE-Cy7	eBioscience/Thermo	Cat#25-0389-42; RRID:AB_1724057
Mouse Anti-CD2 mAb, PE-Cy5 (RPA-2.10)	BD Biosciences	Cat# 555328; RRID:AB_395735
Mouse Anti-CD3 mAb, PE-Cy5 (HIT3a)	BD Biosciences	Cat# 555341; RRID:AB_395747
Mouse Anti-CD4 mAb, PE-Cy5 (RPA-T4)	BD Biosciences	Cat#: 555348; RRID:AB_395753
Mouse Anti-CD7 mAb, PE-Cy5 (M-T701)	BD Biosciences	Cat#: 555362; RRID:AB_395765
Mouse Anti-CD8 mAb, PE-Cy5 (RPA-T8)	BD Biosciences	Cat#: 561951; RRID:AB_10896293
Mouse Anti-CD10 mAb, PE-Cy5 (HI10a)	BD Biosciences	Cat# 555376; RRID:AB_395777
Mouse Anti-CD11b/Mac-1 mAb, PE-Cy5	BD Biosciences	Cat# 555389; RRID:AB_395790
PE/Cyanine5, anti-human CD14 (TuK4)	Biolegend	Cat# 301864; RRID:AB_2860767
Mouse Anti-CD19 mAb, PE-Cy5 (CC2C6)	BD Biosciences	Cat# 555414; RRID:AB_395814
Mouse Anti-CD20 mAb, PE-Cy5 (2H7)	BD Biosciences	Cat# 555624; RRID:AB_395990

Mouse Anti-CD235a mAb, PE-Cy5 (HIR2)	BD Biosciences	Cat# 559944; RRID:AB_397387
Mouse Anti-CD56 mAb, PE-Cy5 (B159)	BD Biosciences	Cat# 561904; RRID:AB_10896277

**Software and algorlithms**		
Graphpad Prism 10 software v10.2.3	GraphPad	RRID:SCR_002798
Flowjo	Flowjo	RRID:SCR_008520
BD FACSDiva	BD Biosciences	RRID:SCR_001456
R software v4.3.3	CRAN	RRID:SCR_001905
