# Supplementary material for: Nonselective β-Adrenergic Receptor Inhibitors Impair Hematopoietic Regeneration in Mice and Humans after Hematopoietic Cell Transplants
Source: Cancer Discov. 2024 Dec 30;15(4):748–66. doi: 10.1158/2159-8290.CD-24-0719 (PMC11962394; doi:10.1158/2159-8290.CD-24-0719)
Supplement: Supplementary Figure 1 — Supplementary Figure S1. Carvedilol and metoprolol do not affect steady-state hematopoiesis. [file cd-24-0719_supplementary_figure_1_suppsf1.pdf]

Supplementary Figure S1

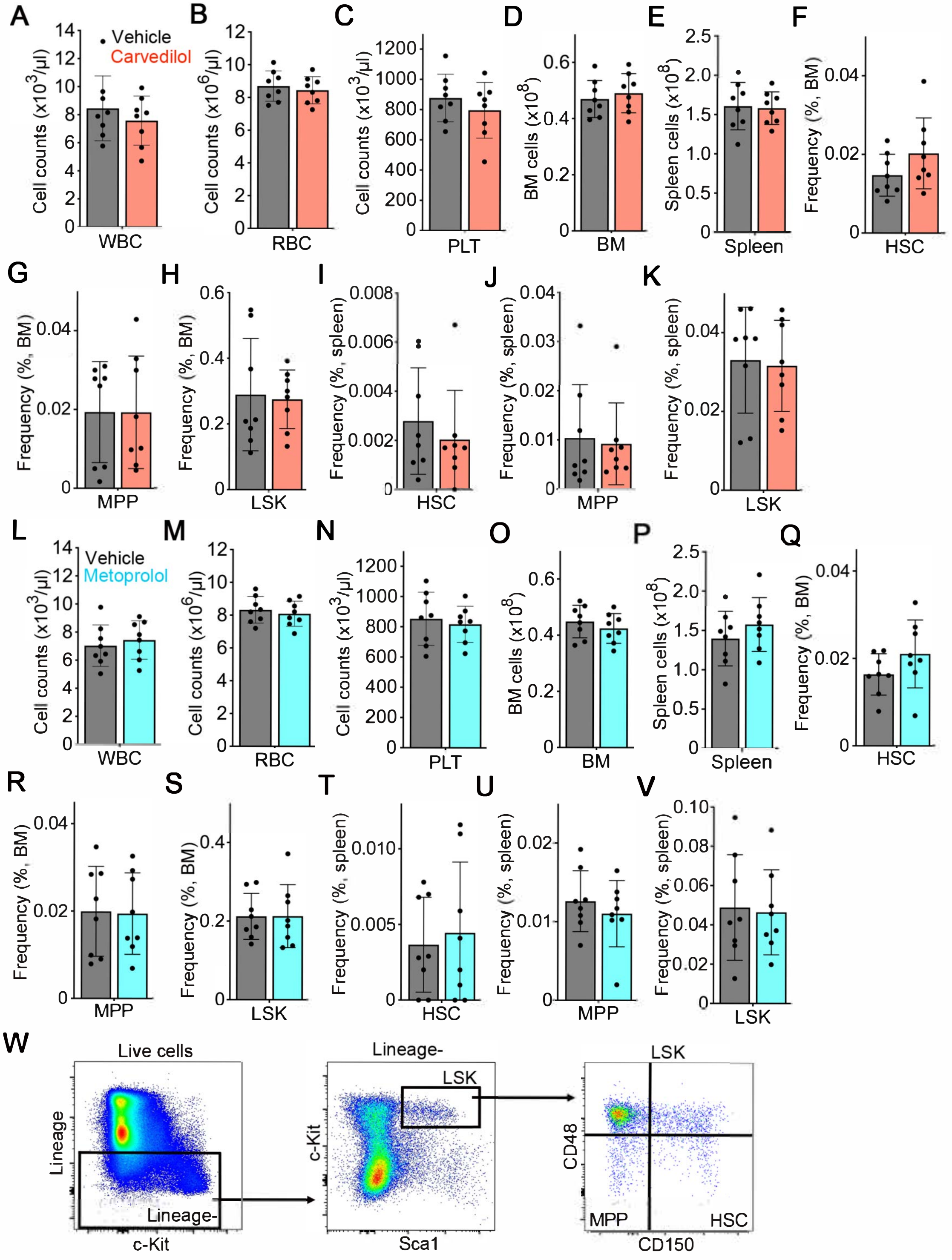

Supplementary Figure S1. Carvedilol and metoprolol do not affect steady-state hematopoiesis. (**A-K**) Mice were treated with carvedilol (a non-selective β adrenergic receptor inhibitor; 20 mg/kg body mass/day) or vehicle (control) for 21 days then hematopoietic parameters were assessed. Each panel shows data from 8 mice per treatment in two independent experiments. Each dot represents a different mouse. All data represent mean ± standard deviation. (**A**-**C**) White blood cell (**A**), red blood cell (**B**), and platelet (**C**) counts. (**D** and **E**) Total bone marrow (**D**) and spleen (**E**) cellularity. (**F-K**) The frequencies of HSCs, MPPs, and LSK cells in the bone marrow (**F-H**) and spleen (**I-K**). (**L-V**) Mice were treated with metoprolol (a β1-selective inhibitor; 20 mg/kg body mass/day) or vehicle (control) for 21 days then hematopoietic parameters were assessed. Each panel shows data from a total of 8 mice per treatment from two independent experiments. The statistical significance of differences among treatments were assessed using two-way ANOVAs followed by Sidak's multiple comparisons adjustments (**A**-**E** and **I**-**S**) or Student's t-tests followed by Holm-Sidak's multiple comparisons adjustments (**F**-**H** and **T**-**V**). All statistical tests were two-sided. (**W**) Flow cytometry gating strategy used to identify HSCs, MPPs, and LSK cells. The markers used to identify each of the cell populations characterized in this study are also listed in Supplementary Table S5.
