# Supplementary material for: Nonselective β-Adrenergic Receptor Inhibitors Impair Hematopoietic Regeneration in Mice and Humans after Hematopoietic Cell Transplants
Source: Cancer Discov. 2024 Dec 30;15(4):748–66. doi: 10.1158/2159-8290.CD-24-0719 (PMC11962394; doi:10.1158/2159-8290.CD-24-0719)
Supplement: Supplementary Figure 2 — Supplementary Figure S2: Metoprolol treatment did not significantly affect hematopoietic regeneration after syngeneic or allogeneic transplantation in mice. [file cd-24-0719_supplementary_figure_2_suppsf2.pdf]

Supplementary Figure S2

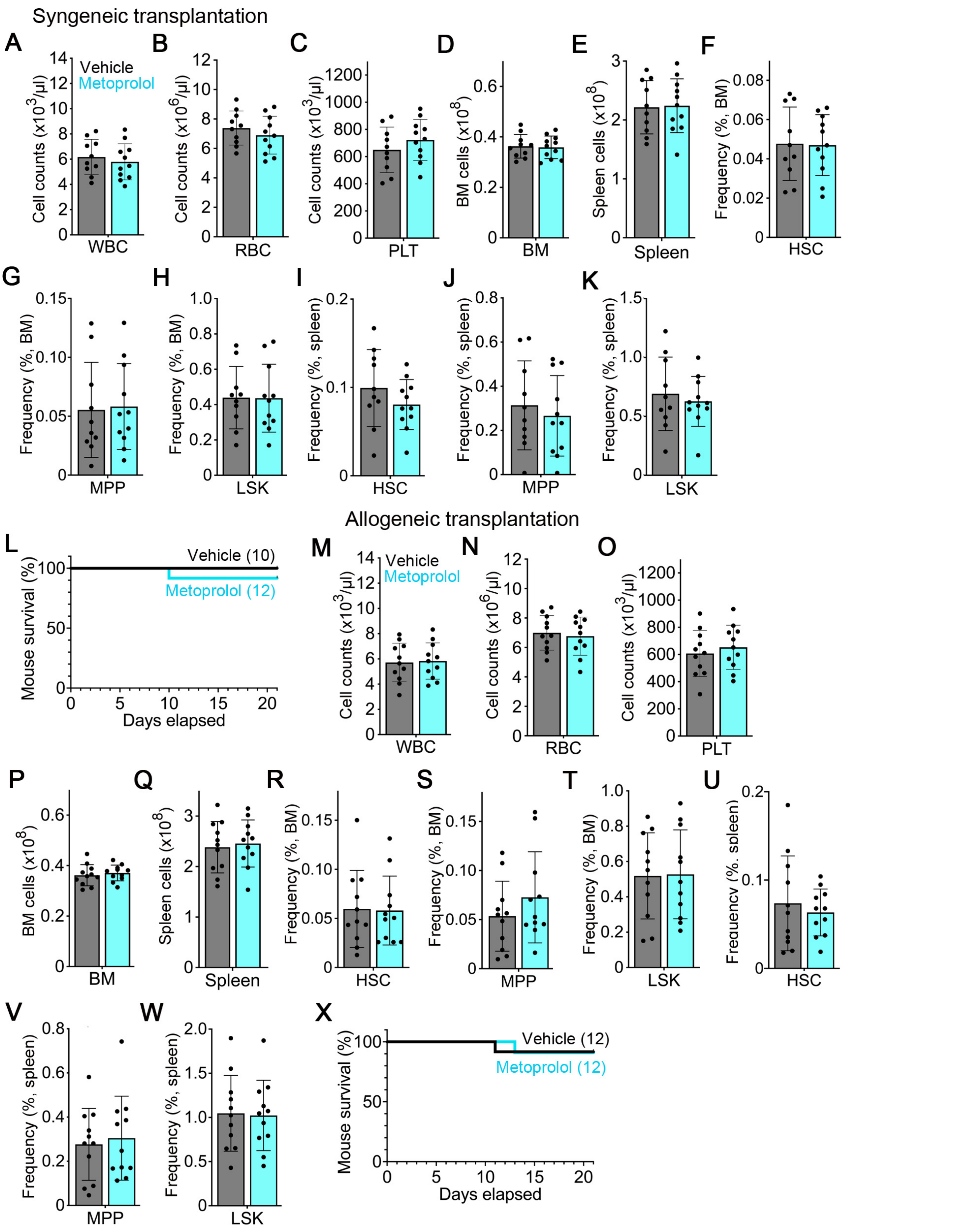

Supplementary Figure S2: Metoprolol treatment did not significantly affect hematopoietic regeneration after syngeneic or allogeneic transplantation in mice. Mice were treated with the β1-selective inhibitor, metoprolol, or vehicle control for 7 days before bone marrow transplantation and 21 days after transplantation. Each panel shows data from two independent experiments and each dot represents a different mouse. All data represent mean ± standard deviation. (**A**-**L**) $6x10^5$ C57BL/Ka bone marrow cells were syngeneically transplanted into irradiated C57BL/Ka-Thy-1.2 recipients. At 21 days after transplantation, we analyzed white blood cell (WBC**, A**), red blood cell (RBC**, B**), and platelet (PLT**, C**) counts as well as bone marrow (**D**) and spleen (**E**) cellularity and the frequencies of HSCs, MPPs, and LSK cells in the bone marrow (**F-H**) and spleen (**I-K**) of metoprolol (blue, n=11) and vehicle (black, n=10) treated mice. (**L**) Survival of metoprolol (blue, n=12) and vehicle (black, n=10) treated mice over time after syngeneic transplantation. (**M**-**X**) $6x10^5$ T cell-depleted LP/J bone marrow cells were allogeneically transplanted into irradiated C57BL/Ka-Thy-1.2 recipients. At 21 days after transplantation, we analyzed blood cell counts (**M**-**O**) as well as bone marrow (**P**) and spleen (**Q**) cellularity and the frequencies of HSCs, MPPs, and LSK cells in the bone marrow (**R**-**T**) and spleen (**U**-**W**) of metoprolol (blue, n=11) and vehicle (black, n=11) treated mice. (**X**) Survival of metoprolol (blue, n=12) and vehicle (black, n=12) treated mice over time after allogeneic transplantation. The statistical significance of differences among treatments were assessed using matched samples two-way ANOVAs followed by Sidak's multiple comparisons adjustments (**A**-**C**, **F**-**H**, **M**-**O**, and **R**-**T**), Student's *t*-tests followed by Holm-Sidak's multiple comparisons adjustments (**D**, **E**, **I**-**K**, **P**, **Q**, and **U**-**W**), or log-rank Mantel-Cox tests (**L** and **X**). All statistical tests were two sided.
