# Supplementary material for: Nonselective β-Adrenergic Receptor Inhibitors Impair Hematopoietic Regeneration in Mice and Humans after Hematopoietic Cell Transplants
Source: Cancer Discov. 2024 Dec 30;15(4):748–66. doi: 10.1158/2159-8290.CD-24-0719 (PMC11962394; doi:10.1158/2159-8290.CD-24-0719)
Supplement: Supplementary Figure 3 — Supplementary Figure S3: Propensity matched analysis of the effect of non-selective b adrenergic receptor inhibitors on hematopoietic regeneration in Vanderbilt patients undergoing autologous and allogeneic transplantation. [file cd-24-0719_supplementary_figure_3_suppsf3.pdf]

Supplementary Figure S3

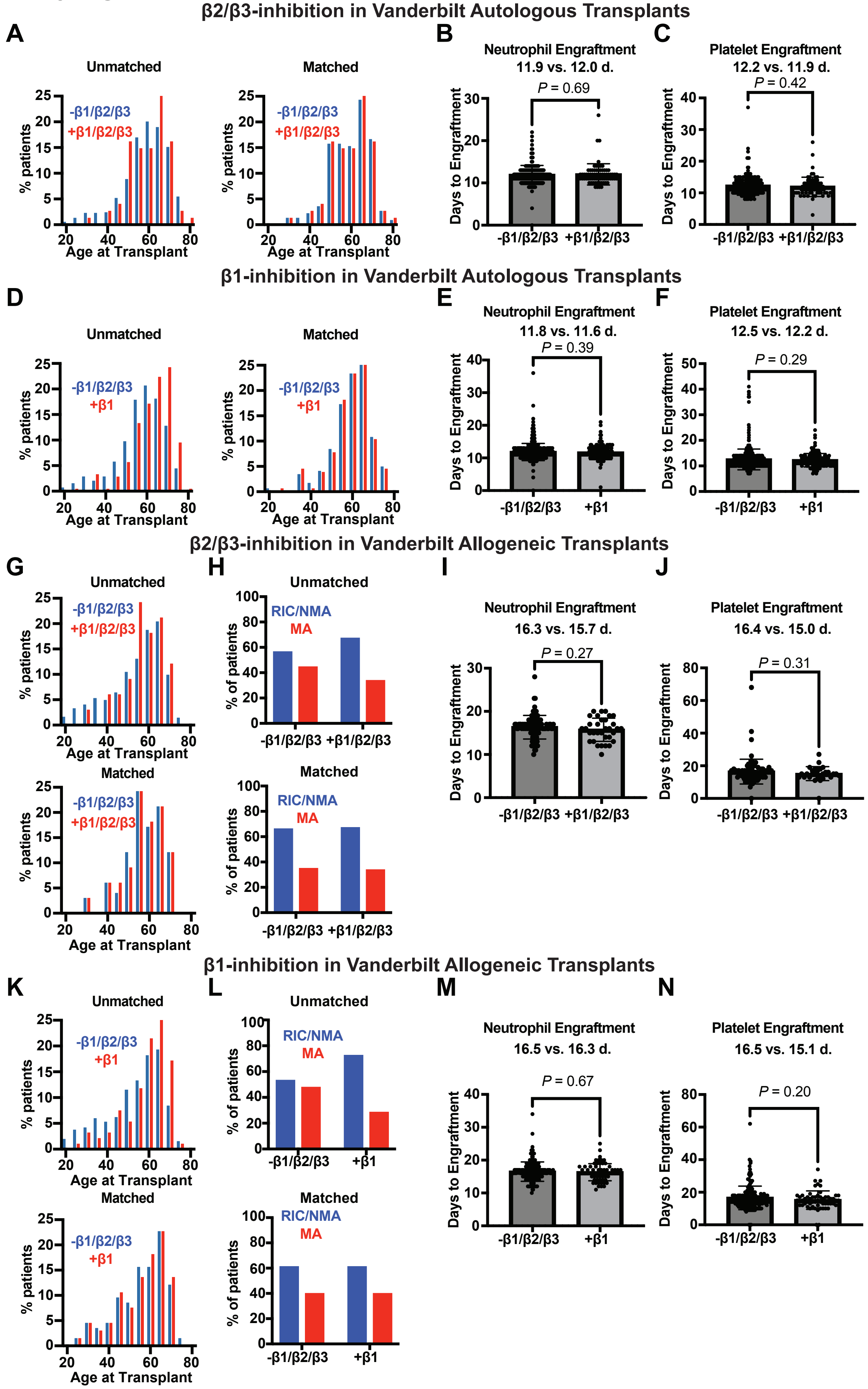

Supplementary Figure S3: Propensity matched analysis of the effect of non-selective β adrenergic receptor inhibitors on hematopoietic regeneration in Vanderbilt patients undergoing autologous and allogeneic transplantation. (A) Propensity matching for age in Vanderbilt autologous transplant recipients who were (*n*=74), or were not (*n*=1,037), treated with non-selective β blockers. Time to neutrophil **(B)** and platelet **(C)** engraftment in matched groups (*n*=74 and 222 after 1:3 matching) is shown. **(D)** Propensity matching for age in Vanderbilt autologous transplant patients who were (*n*=210), or were not (*n*=827), treated with β1-selective inhibitors. Time to neutrophil **(E)** and platelet **(F)** engraftment in matched groups (*n*=154 and 462 after 1:3 matching). Propensity matching for age **(G)** and conditioning regimen **(H)** in Vanderbilt allogeneic transplant patients who were (*n*=33), or were not (*n*=543), treated with non-selective β blockers (RIC means reduced intensity conditioning, NMA/MA means non-myeloablative versus myeloablative conditioning). Time to neutrophil **(I)** and platelet **(J)** engraftment in matched groups (*n*=33 and 99 after 1:3 matching). Propensity matching for age **(K)** and conditioning regimen **(L)** in Vanderbilt allogeneic transplant patients who were treated with β1-selective inhibitors (*n*=93) versus no b blocker (*n*=450). Time to neutrophil **(M)** and platelet **(N)** engraftment in matched (*n*=66 and 198 after 1:3 matching). The statistical significance of differences among groups was assessed using Student's *t* tests. All data represent mean ± standard deviation.
