# Supplementary material for: Nonselective β-Adrenergic Receptor Inhibitors Impair Hematopoietic Regeneration in Mice and Humans after Hematopoietic Cell Transplants
Source: Cancer Discov. 2024 Dec 30;15(4):748–66. doi: 10.1158/2159-8290.CD-24-0719 (PMC11962394; doi:10.1158/2159-8290.CD-24-0719)
Supplement: Supplementary Figure 4 — Supplementary Figure S4: Clinical variables associated with time to hematopoietic regeneration after autologous transplantation at UTSW. [file cd-24-0719_supplementary_figure_4_suppsf4.pdf]

Supplementary Figure S4

UT Southwestern Autologous Transplants

A Time to Neutrophil Engraftment

Variable	Condition (*n*)	Coefficient (B) (95% CI)	p-value
Age	All (850)	0.01 (0, 0.02)	0.042
β-blocker	None (666)	reference	
	β1 (118)	0.03 (-0.28, 0.34)	0.86
	β1/β2/β3 (66)	0.53 (0.13, 0.93)	0.009

B Time to Platelet Engraftment

Variable	Condition (*n*)	Coefficient (B) (95% CI)	p-value
Age	All (850)	0.02 (-0.01, 0.05)	0.27
β-blocker	None (666)	reference	
	β1 (118)	-1.1 (-2.3, 0.06)	0.063
	β1/β2/β3 (66)	0.08 (-1.5, 1.6)	0.92

Supplementary Figure S4: Clinical variables associated with time to hematopoietic regeneration after autologous transplantation at UTSW. A single-variable regression analysis was performed to identify clinical variables associated with changes in time to neutrophil (**A**) or platelet (**B**) engraftment after autologous HCT at UTSW. Risk factor coefficients (*B*) reflect the change in number of days to engraftment per unit of each predictive variable, with units being per year for age and binary (yes/no) for all other variables. *B* ± 95% confidence intervals is shown. Positive *B* values reflect delayed engraftment.
