# Supplementary material for: Nonselective β-Adrenergic Receptor Inhibitors Impair Hematopoietic Regeneration in Mice and Humans after Hematopoietic Cell Transplants
Source: Cancer Discov. 2024 Dec 30;15(4):748–66. doi: 10.1158/2159-8290.CD-24-0719 (PMC11962394; doi:10.1158/2159-8290.CD-24-0719)
Supplement: Supplementary Figure 5 — Supplementary Figure S5: Clinical variables associated with time to hematopoietic regeneration after allogeneic transplantation at UTSW. [file cd-24-0719_supplementary_figure_5_suppsf5.pdf]

Supplementary Figure S5

A

UT Southwestern Allogeneic Transplant
Time to Neutrophil Engraftment

Variable	Condition (*n*)	Coefficient (*B*) (95% CI)	p-value
**β-blocker**	None (237)	Reference	
	β1 (23)	1.4	0.086
	β1/β2/β3 (14)	4.1	<0.001
**Conditioning**	MAC (116)	Reference	
	RIC/NMA (158)	1.4	<0.001
**β–agonist**	No (241)	Reference	
	Yes (33)	0.24	0.74
**Cell number**	(250)	0	0.26
**GvHD treatment**	None (9)	Reference	
	MTX (104)	−1.6	0.23
	PTCy (161)	0.030	0.98
**Disease**	Lymphoid (78)	Reference	
	Myeloid (195)	1.7	<0.001
**Age at Transplant (years)**	(274)	0.060	<0.001
**Cell source**	BM (36)	Reference	
	PB (238)	1.7	0.014
**Donor match**	MRD/MUD (210)	Reference	
	MMUD (19)	1.6	0.078
	Haplo (45)	−0.030	0.96
**CMV infection**	No (198)	Reference	
	Yes (76)	−1.4	0.005
**Acute GvHD**	No (100)	Reference	
	Yes (174)	−0.13	0.79
**Ferritin**	(231)	0	0.15
**Splenomegaly**	No (208)	Reference	
	Yes (66)	0.68	0.21
**Infection**	No (122)	Reference	
	Yes (152)	−0.85	0.067

B

UT Southwestern Allogeneic Transplant
Time to Platelet Engraftment

Variable	Condition (*n*)	Coefficient (*B*) (95% CI)	p-value
**β-blocker**	None (237)	Reference	
	β1 (23)	2.6	0.32
	β1/β2/β3 (14)	13	<0.001
**Conditioning**	MAC (116)	Reference	
	RIC/NMA (158)	3.3	0.025
**β–agonist**	No (241)	Reference	
	Yes (33)	2.0	0.38
**Cell number**	(250)	0	0.48
**GvHD treatment**	None (9)	Reference	
	MTX (104)	−0.31	0.94
	PTCy (161)	3.6	0.39
**Disease**	Lymphoid (78)	Reference	
	Myeloid (195)	4.2	0.010
**Age at Transplant (years)**	(274)	0.16	<0.001
**Cell source**	BM (36)	Reference	
	PB (238)	1.2	0.60
**Donor match**	Well (210)	Reference	
	MMUD (19)	6.6	0.022
	Haplo (45)	2.1	0.29
**CMV infection**	No (198)	Reference	
	Yes (76)	−4.4	0.007
**Acute GvHD**	No (100)	Reference	
	Yes (174)	1.8	0.23
**Ferritin**	(231)	0	0.49
**Splenomegaly**	No (208)	Reference	
	Yes (66)	0.63	0.72
**Infection**	No (122)	Reference	
	Yes (152)	−1.3	0.38

Supplementary Figure S5: Clinical variables associated with time to hematopoietic regeneration after allogeneic transplantation at UTSW. A single-variable regression analysis was performed to identify clinical variables associated with changes in time to neutrophil (**A**) and platelet (**B**) engraftment after allogeneic HCT at UTSW. Risk factor coefficients (*B*) reflect the change in number of days to engraftment per unit of each predictive variable, with units being per year for age, ng/ml for ferritin, and binary (yes/no) for all other variables. *B* ± 95% confidence intervals is shown. Positive *B* values reflect delayed engraftment.
