# Supplementary material for: Nonselective β-Adrenergic Receptor Inhibitors Impair Hematopoietic Regeneration in Mice and Humans after Hematopoietic Cell Transplants
Source: Cancer Discov. 2024 Dec 30;15(4):748–66. doi: 10.1158/2159-8290.CD-24-0719 (PMC11962394; doi:10.1158/2159-8290.CD-24-0719)
Supplement: Supplementary Figure 6 — Supplementary Figure S6: Clinical variables associated with time to hematopoietic regeneration after autologous transplantation in Vanderbilt patients. [file cd-24-0719_supplementary_figure_6_suppsf6.pdf]

Supplementary Figure S6

Vanderbilt Autologous Transplants

A Time to Neutrophil Engraftment

Variable	Condition (*n*)	Coefficient (B) (95% CI)	p-value
Age	All (1109)	0.01 (0, 0.02)	0.11
β-blocker	None (825)	reference	
	β1 (210)	-0.15 (-0.52, 0.22)	0.42
	β1/β2/β3 (74)	0.21 (-0.36, 0.79)	0.46

B Time to Platelet Engraftment

Variable	Condition (*n*)	Coefficient (B) (95% CI)	p-value
Age	All (1109)	0.09 (-0.03, 0.21)	0.15
β-blocker	None (825)	reference	
	β1 (210)	-1.3 (-4.7, 2.1)	0.46
	β1/β2/β3 (74)	-1.4 (-6.7, 3.9)	0.61

Supplementary Figure S6: Clinical variables associated with time to hematopoietic regeneration after autologous transplantation in Vanderbilt patients. A single-variable regression analysis was performed to identify clinical variables associated with changes in time to neutrophil (**A**) or platelet (**B**) engraftment after autologous HCT at Vanderbilt. Risk factor coefficients (*B*) reflect the change in number of days to engraftment per unit of each predictive variable, with units being per year for age and binary (yes/no) for all other variables. *B* ± 95% confidence intervals is shown. Positive *B* values reflect delayed engraftment.
