# Supplementary material for: Nonselective β-Adrenergic Receptor Inhibitors Impair Hematopoietic Regeneration in Mice and Humans after Hematopoietic Cell Transplants
Source: Cancer Discov. 2024 Dec 30;15(4):748–66. doi: 10.1158/2159-8290.CD-24-0719 (PMC11962394; doi:10.1158/2159-8290.CD-24-0719)
Supplement: Supplementary Figure 7 — Supplementary Figure S7: Clinical variables associated with time to hematopoietic regeneration after allogeneic transplantation at Vanderbilt. [file cd-24-0719_supplementary_figure_7_suppsf7.pdf]

Supplementary Figure S7

A

Vanderbilt Allogeneic Transplant
Time to Neutrophil Engraftment

Variable	Condition (*n*)	Coefficient (*B*) (95% CI)	p-value
**β-blocker**	None (405)	Reference	
	β1 (76)	−0.26	0.48
	β1/β2/β3 (30)	−0.9	0.10
**Conditioning**	MAC (241)	Reference	
	RIC/NMA (270)	0.48	0.063
**Disease**	AML (250)	Reference	
	CML (35)	1.3	0.017
	MPN (15)	0.33	0.67
	MDS (206)	0.63	0.022
**Cell number**	(489)	−0.04	0.53
**Age at Transplant (years)**	(511)	0	0.80
**Cell source**	BM (26)	Reference	
	PB (485)	−1.7	0.004
**Donor match**	MRD/MUD (489)	Reference	
	MMUD (22)	0.03	0.97
**Acute GvHD**	No (213)	Reference	
	Yes (281)	0.54	0.35
**GvHD treatment**	None (256)	Reference	
	MTX (241)	−0.33	0.23
	PTCy (9)	3.4	<0.001
	unknown (5)	−1.4	0.30
**CMV serostatus**	low risk (140)	Reference	
	intermediate (240)	0.5	0.11
	high risk (131)	0.1	0.78

B

Vanderbilt Allogeneic Transplant
Time to Platelet Engraftment

Variable	Condition (*n*)	Coefficient (*B*) (95% CI)	p-value
**β-blocker**	None (405)	Reference	
	β1 (76)	2.1	0.38
	β1/β2/β3 (30)	11	0.003
**Conditioning**	MAC (241)	Reference	
	RIC/NMA (270)	−4.3	0.013
**Disease**	AML (250)	Reference	
	CML (35)	−0.13	0.97
	MPN (15)	0.17	0.97
	MDS (206)	1.3	0.48
**Cell number**	(489)	−0.6	0.18
**Age at Transplant (years)**	(511)	−0.07	0.30
**Cell source**	BM (26)	Reference	
	PB (485)	−9.7	0.013
**Donor match**	MRD/MUD (489)	Reference	
	MMUD (22)	19	<0.001
**Acute GvHD**	No (213)	Reference	
	Yes (281)	2.6	0.16
**GvHD treatment**	None (256)	Reference	
	MTX (241)	4.2	0.016
	PTCy (9)	17	0.010
	unknown (5)	2.5	0.78
**CMV serostatus**	low risk (140)	Reference	
	intermediate (240)	3.5	0.087
	high risk (131)	−0.27	0.91

Supplementary Figure S7: Clinical variables associated with time to hematopoietic regeneration after allogeneic transplantation at Vanderbilt. A single-variable regression analysis was performed to identify clinical variables associated with changes in time to neutrophil (**A**) and platelet (**B**) engraftment after allogeneic HCT at Vanderbilt. Risk factor coefficients (*B*) reflect the change in number of days to engraftment per unit of each predictive variable, with units being per year for age, and binary (yes/no) for all other variables. *B* ± 95% confidence intervals is shown. Positive *B* values reflect delayed engraftment.
