# Supplementary material for: Nonselective β-Adrenergic Receptor Inhibitors Impair Hematopoietic Regeneration in Mice and Humans after Hematopoietic Cell Transplants
Source: Cancer Discov. 2024 Dec 30;15(4):748–66. doi: 10.1158/2159-8290.CD-24-0719 (PMC11962394; doi:10.1158/2159-8290.CD-24-0719)
Supplement: Supplementary Figure 8 — Supplementary Figure S8: Hematopoietic stem and progenitor cell frequencies after allogeneic transplantation. [file cd-24-0719_supplementary_figure_8_suppsf8.pdf]

Supplementary Figure S8

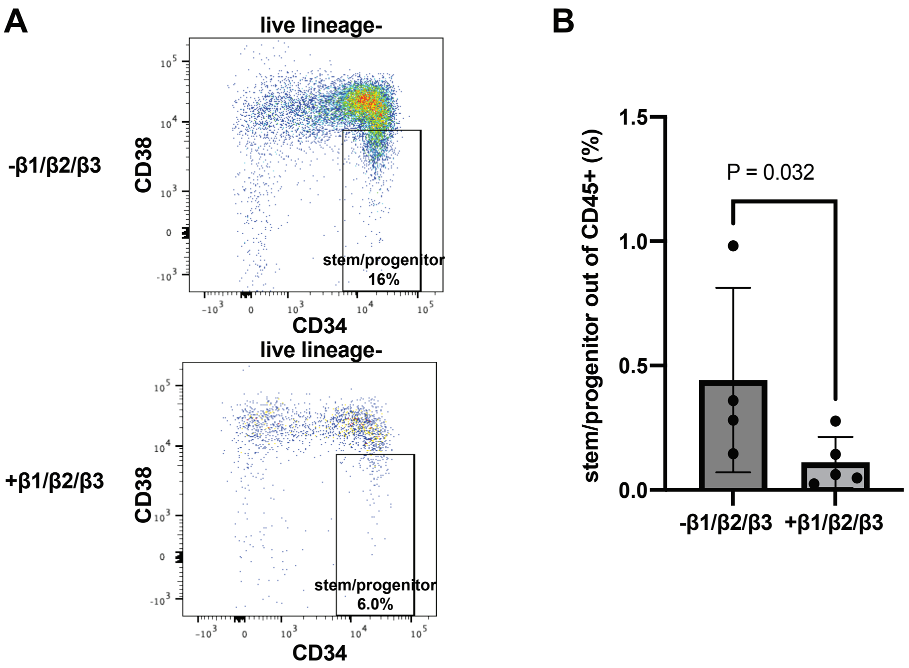

Supplementary Figure S8: Hematopoietic stem and progenitor cell frequencies after allogeneic transplantation. Bone marrow specimens were obtained at 100 days after allogeneic HCT from patients on non-selective β blockers and age-matched controls (n=5 and 4, mean ages 59 and 63, respectively). (**A**) Representative flow cytometry gating to identify Lineage^{-}CD34^{+}CD38^{-} stem/progenitor cells. (**B**) Frequencies of Lineage^{-}CD34^{+}CD38^{-} stem/progenitor cells as a percentage of all live CD45^{+} bone marrow cells. All data represent mean ± standard deviation. The statistical significance of differences between groups was assessed using a Mann-Whitney *U* test.
