# Supplementary material for: Nonselective β-Adrenergic Receptor Inhibitors Impair Hematopoietic Regeneration in Mice and Humans after Hematopoietic Cell Transplants
Source: Cancer Discov. 2024 Dec 30;15(4):748–66. doi: 10.1158/2159-8290.CD-24-0719 (PMC11962394; doi:10.1158/2159-8290.CD-24-0719)
Supplement: Supplementary Figure 9 — Supplementary Figure S9: The inhibitory effect of carvedilol on hematopoietic regeneration after syngeneic transplantation can be overcome by transplanting larger doses of bone marrow cells. [file cd-24-0719_supplementary_figure_9_suppsf9.pdf]

Supplementary Figure S9

Syngeneic transplantation

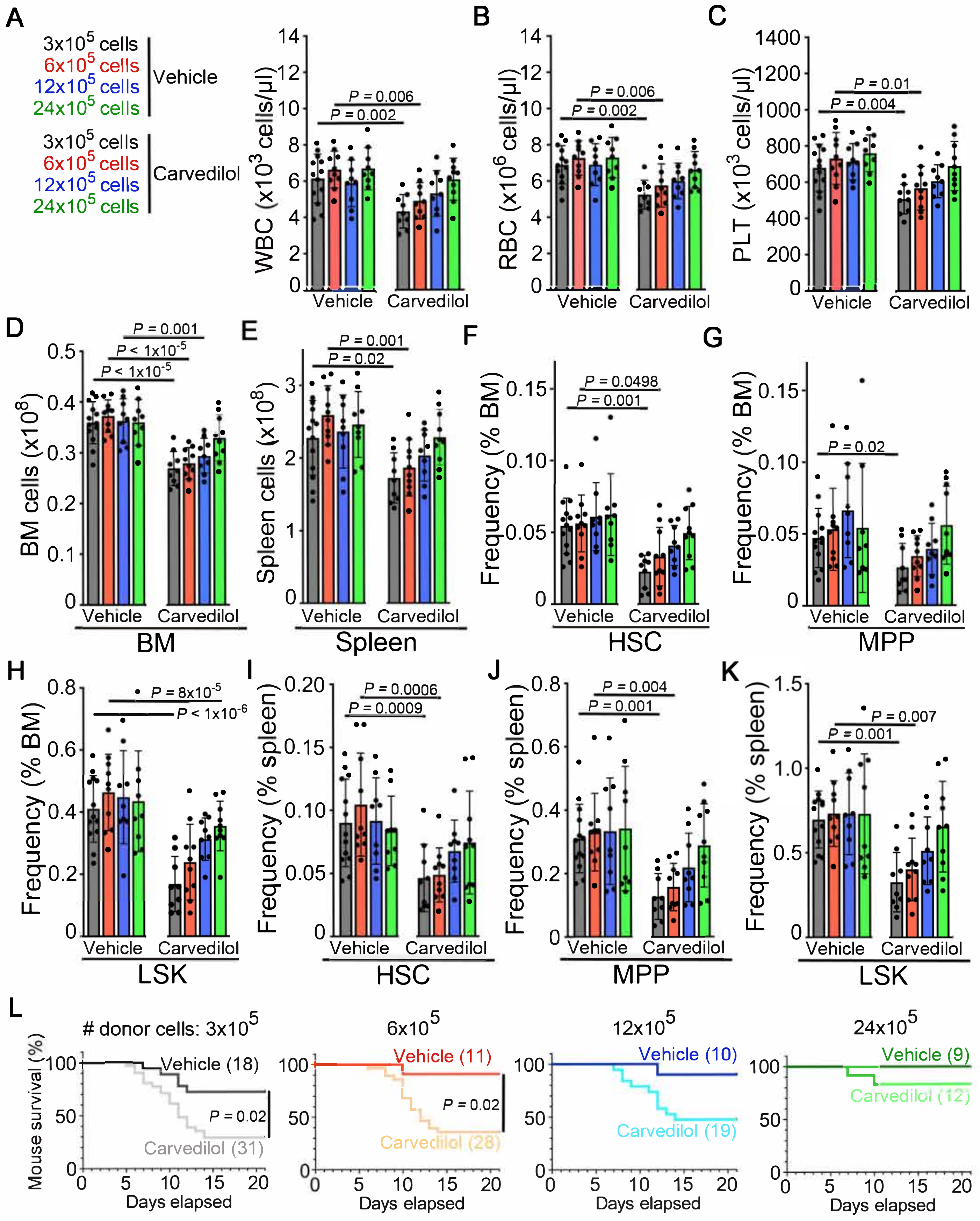

Supplementary Figure S9: The inhibitory effect of carvedilol on hematopoietic regeneration after syngeneic transplantation can be overcome by transplanting larger doses of bone marrow cells. Mice were treated with carvedilol or vehicle for 7 days before and 21 days after transplantation. We transplanted $3x10^5$, $6x10^5$, $12x10^5$, or $24x10^5$ C57BL/Ka bone marrow cells into irradiated C57BL/Ka-Thy-1.2 recipients. Each panel shows data from three independent experiments. Panels **A-K** have 9 to 13 recipients per treatment. Each dot represents a different mouse. All data represent mean ± standard deviation. (**A**-**C**) White blood cell (**A**), red blood cell (**B**), and platelet (**C**) counts from carvedilol (right) or vehicle (left) treated mice 21 days after transplantation. (**D** and **E**) Total bone marrow (**D**) and spleen (**E**) cellularity. (**F**-**K**) The frequencies of HSCs, MPPs, and LSK cells in the bone marrow (**F-H**) and spleen (**I-K**). (**L**) Survival of carvedilol and vehicle treated mice over time after syngeneic transplantation. The numbers of mice per treatment are shown in each panel. The statistical significance of differences among treatments were assessed using two-way ANOVAs followed by Sidak's multiple comparisons adjustments (**A-E**, **G-I** and **K**), Student's t-test followed by Holm-Sidak's multiple comparisons adjustment (**F**, **J**) or log-rank Mantel-Cox tests followed by Holm-Sidak's multiple comparisons adjustment (**L**). All statistical tests were two sided. When statistical differences are significant (P<0.05), P values are presented as exact values.
