# Supplementary material for: Nonselective β-Adrenergic Receptor Inhibitors Impair Hematopoietic Regeneration in Mice and Humans after Hematopoietic Cell Transplants
Source: Cancer Discov. 2024 Dec 30;15(4):748–66. doi: 10.1158/2159-8290.CD-24-0719 (PMC11962394; doi:10.1158/2159-8290.CD-24-0719)
Supplement: Supplementary Figure 10 — Supplementary Figure S10: Post-transplant chemotherapy or use of non-selective b blockers was not associated with changes in neutrophil engraftment after allogeneic transplantation in Vanderbilt allogeneic HCT patients. [file cd-24-0719_supplementary_figure_10_suppsf10.pdf]

Supplementary Figure S10

Vanderbilt Allogeneic Transplants

A Time to Neutrophil Engraftment without MTX/PTCy

Variable		(*n*)	Coefficient (*B*) (95% CI)	p–value
**β-blocker**	none	192	Reference	
	β1	22	−0.44 (−1.6, 0.76)	0.47
	β1/β2/β3	10	−1.1 (−2.8, 0.67)	0.23
**Conditioning**	MAC	202	Reference	
	RIC/NMA	22	1.7 (0.31, 3.0)	0.017
**Disease**	AML	134	Reference	
	CML	19	0.48 (−0.87, 1.8)	0.49
	MPN	7	1.1 (−1.0, 3.1)	0.32
	MDS	64	0.60 (−0.23, 1.4)	0.16
**Cell number**		224	−0.19 (−0.39, 0)	0.055
**Age at transplant (years)**		224	−0.010 (−0.04, 0.03)	0.72
**Cell source**	BM	3	Reference	
	PB	221	2.0 (−1.1, 5.1)	0.21
**Donor match**	MRD/MUD	209	Reference	
	MMUD	15	0.26 (−1.2, 1.7)	0.73
**aGVHD**	no	99	Reference	
	yes	125	1.1 (0.37, 1.8)	0.003
**CMV serostat.**	low risk	64	Reference	
	intermediate	111	0.40 (−0.43, 1.2)	0.34
	high risk	49	0.51 (−0.5, 1.52)	0.32

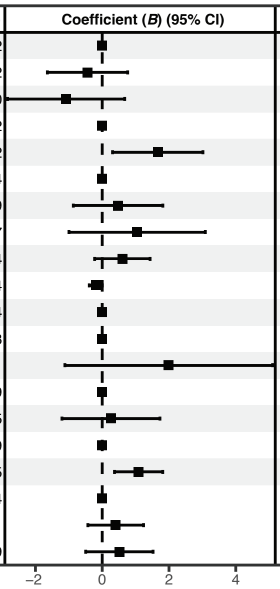

B Time to Neutrophil Engraftment with MTX/PTCy

Variable		(*n*)	Coefficient (*B*) (95% CI)	p–value
**β-blocker**	none	181	Reference	
	β1	46	−0.49 (−1.4, 0.45)	0.31
	β1/β2/β3	17	−0.87 (−2.3, 0.59)	0.25
**Conditioning**	MAC	8	Reference	
	RIC/NMA	236	1.7 (−0.49, 4.0)	0.13
**Disease**	AML	94	Reference	
	CML	11	1.3 (−0.55, 3.1)	0.18
	MPN	8	−0.15 (−2.2, 1.9)	0.89
	MDS	131	0.97 (0.18, 1.8)	0.017
**Cell number**		244	0.16 (−0.04, 0.35)	0.13
**Age at transplant (years)**		244	0 (−0.06, 0.05)	0.89
**Cell source**	BM	1	Reference	
	PB	243	0.98 (−4.7, 6.7)	0.74
**Donor match**	MRD/MUD	240	Reference	
	MMUD	4	0.39 (−2.5, 3.3)	0.79
**aGVHD**	no	108	Reference	
	yes	136	−0.13 (−0.88, 0.63)	0.75
**CMV serostat.**	low risk	66	Reference	
	intermediate	111	0.18 (−0.72, 1.1)	0.70
	high risk	67	−0.55 (−1.5, 0.44)	0.28

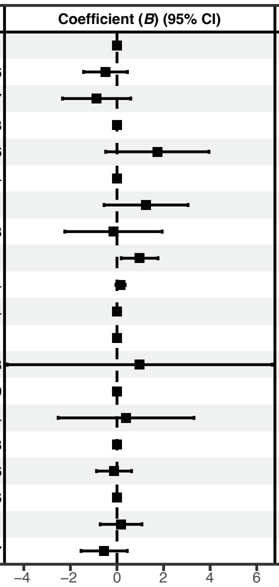

Supplementary Figure S10: Post-transplant chemotherapy or use of non-selective β blockers was not associated with changes in neutrophil engraftment after allogeneic transplantation in Vanderbilt allogeneic HCT patients. The risk factor coefficients (*B*) for time to neutrophil engraftment among Vanderbilt patients who received allogeneic HCTs and who did not (**A**) or did (**B**) receive myelosuppressive graft-versus-host disease prophylaxis with methotrexate or post-transplant cyclophosphamide. A generalized linear model was used, with covariates including β1-selective inhibitor use, non-selective β blocker use, conditioning regimen (myeloablative conditioning [MAC] or reduced-intensity conditioning/non-myeloablative conditioning [RIC/NMA]), underlying disease (acute myeloid leukemia [AML], chronic myeloid leukemia [CML], myeloproliferative neoplasm [MPN], or myelodysplastic syndrome [MDS]), cell dose ($CD34^+$ cells/kg), age, cell source (bone marrow [BM] or peripheral blood [PB]), donor matching (matched related/matched unrelated donor [MRD/MUD], mismatched unrelated donor [MMUD], or haploidentical [Haplo]), acute graft-versus-host disease, and CMV serostatus (low risk = donor - / recipient -, intermediate risk = donor + / recipient + or -, high risk = donor - / receipient +). *B* reflects the number of additional days required for neutrophil engraftment per unit of each predictive variable, with units being per year for age and binary (yes/no) for all other variables. *B* ± 95% confidence intervals is shown. The dashed vertical line represents *B* of 0.
