# Supplementary material for: Nonselective β-Adrenergic Receptor Inhibitors Impair Hematopoietic Regeneration in Mice and Humans after Hematopoietic Cell Transplants
Source: Cancer Discov. 2024 Dec 30;15(4):748–66. doi: 10.1158/2159-8290.CD-24-0719 (PMC11962394; doi:10.1158/2159-8290.CD-24-0719)
Supplement: Supplementary Figure 11 — Supplementary Figure S11: Infection, graft-versus-host disease, and causes of death in UTSW allogeneic HCT recipients. [file cd-24-0719_supplementary_figure_11_suppsf11.pdf]

Supplementary Figure S11

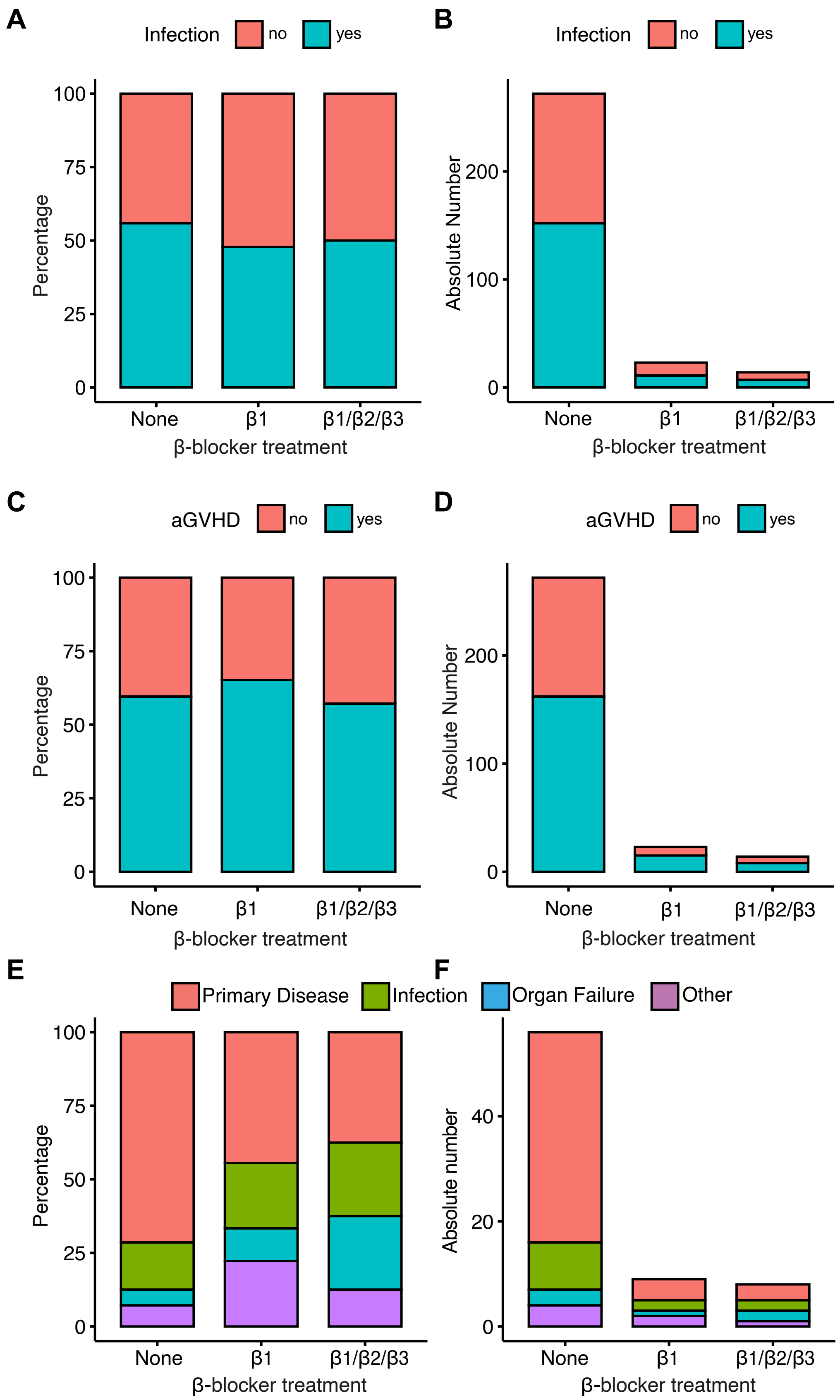

Supplementary Figure S11: Infection, graft-versus-host disease, and causes of death in UTSW allogeneic HCT recipients. The frequency (**A**) and absolute number (**B**) of patients who received β1-selective inhibitors, non-selective β blockers, or no β blockers who developed clinically significant infections. The frequency (**C**) and absolute number (**D**) of patients who received β1-selective inhibitors, non-selective β blockers, or no β blocker who developed acute graft-versus-host disease (aGvHD) of any grade. The frequency (**E**) and absolute number (**F**) of causes of death. The statistical significance of differences between groups was assessed using Chi squared tests: There was a significant increase in infections or organ failure, combined, as a cause of death in patients on non-selective β blockers as compared to patients not treated with β blockers (29% versus 5.5%, *P*=0.0029). No other significant differences were observed.
