# Supplementary material for: Nonselective β-Adrenergic Receptor Inhibitors Impair Hematopoietic Regeneration in Mice and Humans after Hematopoietic Cell Transplants
Source: Cancer Discov. 2024 Dec 30;15(4):748–66. doi: 10.1158/2159-8290.CD-24-0719 (PMC11962394; doi:10.1158/2159-8290.CD-24-0719)
Supplement: Supplementary Figure 12 — Supplementary Figure S12: Infection, graft-versus-host disease, and causes of death in Vanderbilt allogeneic HCT recipients. [file cd-24-0719_supplementary_figure_12_suppsf12.pdf]

## Supplementary Figure S12

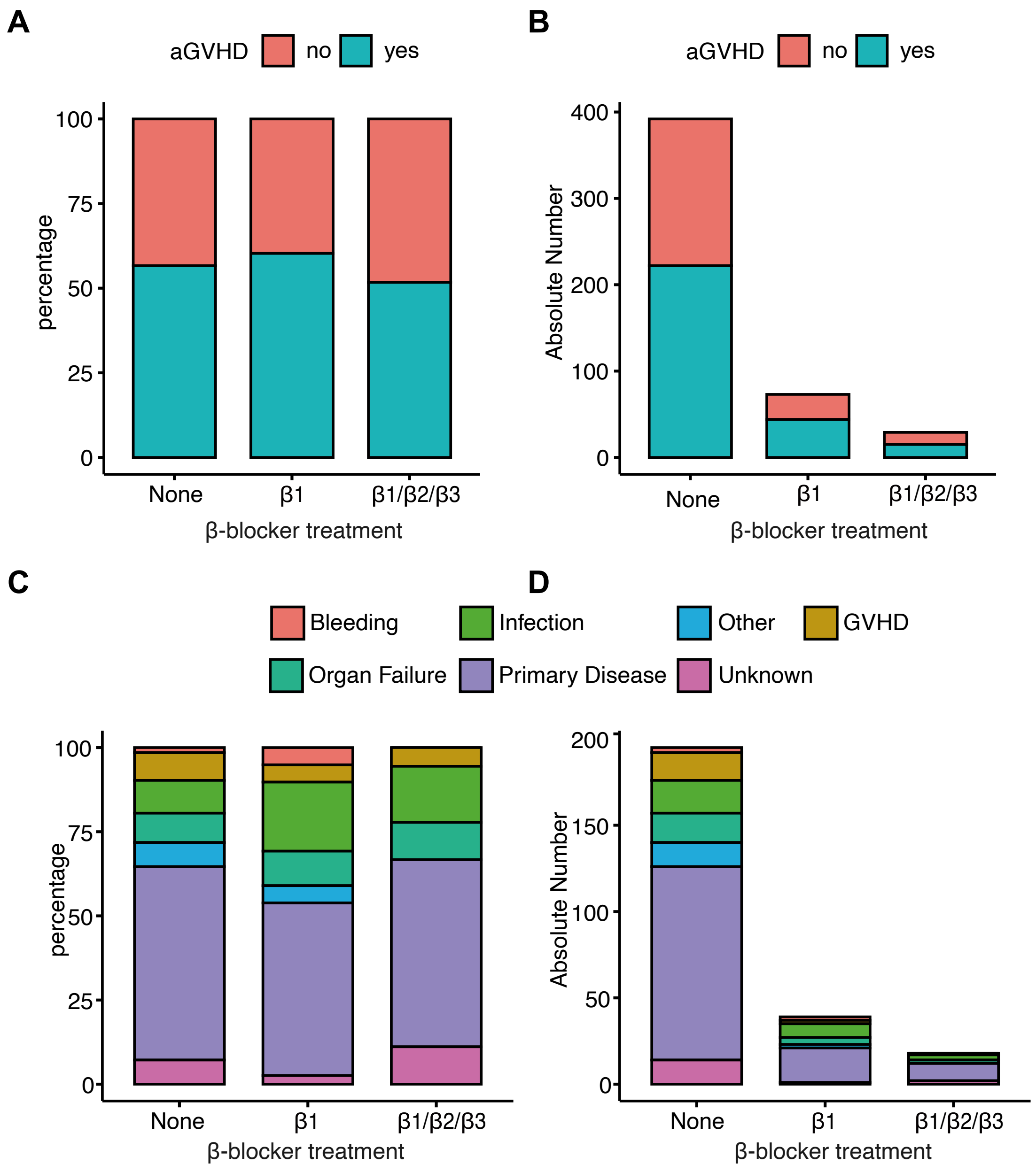


**Supplementary Figure S12: Infection, graft-versus-host disease, and causes of death in Vanderbilt allogeneic HCT recipients.** The frequency (**A**) and absolute number (**B**) of patients who received β1-selective inhibitors, non-selective β blockers, or no β blockers who developed acute graft-versus-host disease (aGvHD) of any grade. The frequency (**C**) and absolute number (**D**) of causes of death. The statistical significance of differences between groups was assessed using Chi squared tests: no significant differences were found.
