# Supplementary material for: Nonselective β-Adrenergic Receptor Inhibitors Impair Hematopoietic Regeneration in Mice and Humans after Hematopoietic Cell Transplants
Source: Cancer Discov. 2024 Dec 30;15(4):748–66. doi: 10.1158/2159-8290.CD-24-0719 (PMC11962394; doi:10.1158/2159-8290.CD-24-0719)
Supplement: Supplementary Figure 13 — Supplementary Figure S13: Discontinuation of b blockers around the time of transplantation rescues hematopoietic regeneration. [file cd-24-0719_supplementary_figure_13_suppsf13.pdf]

Supplementary Figure S13

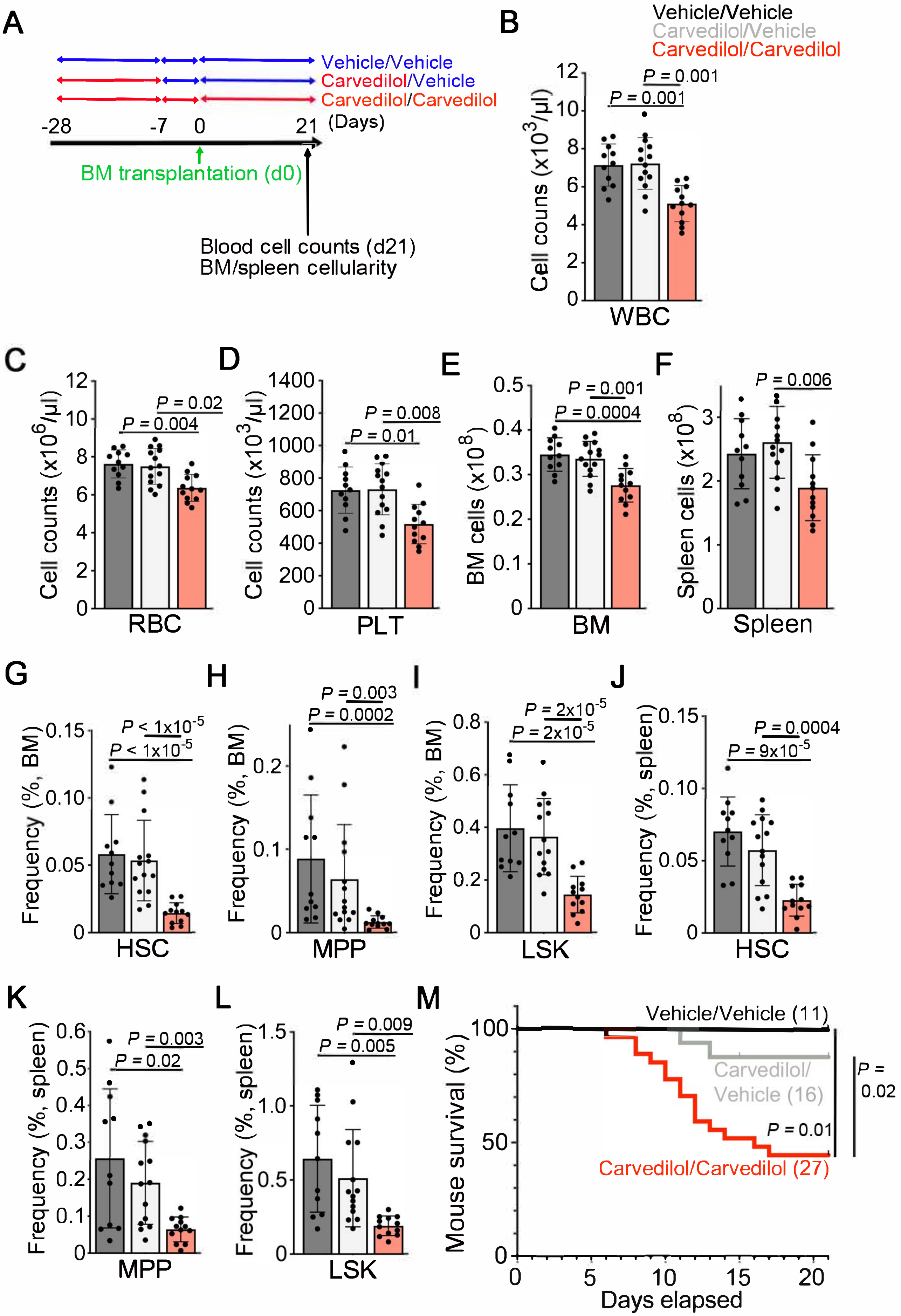

Supplementary Figure S13: Discontinuation of β blockers around the time of transplantation rescues hematopoietic regeneration. (**A**) Mice were treated with carvedilol or vehicle for 28 days before and 21 days after syngeneic transplantation. In a third treatment arm, mice received carvedilol from 28 to 7 days before transplantation and then were switched to vehicle until 21 days after transplantation. We transplanted $6x10^5$ C57BL/Ka bone marrow cells into irradiated C57BL/Ka-Thy-1.2 recipients. Each panel shows data from three independent experiments. Panels **B-L** have a total of 11 to 14 recipients per treatment. Each dot represents a different mouse. All data represent mean ± standard deviation. (**B**-**D**) White blood cell (**B**), red blood cell (**C**), and platelet (**D**) counts at 21 days after transplantation. (**E**, **F**) Total bone marrow (**E**) and spleen (**F**) cellularity. (**G**-**L**) The frequencies of HSCs, MPPs, and LSK cells in the bone marrow (**G-I**) and spleen (**J-L**). (**M**) Survival of carvedilol and vehicle-treated mice over time after transplantation. The statistical significance of differences among treatments were assessed using matched samples two-way ANOVAs followed by Sidak's multiple comparisons adjustments (**B**-**D**), one-way ANOVAs followed by Tukey's multiple comparisons adjustments (**E-I**), Welch's one-way ANOVAs followed by Dunnett's T3 multiple comparisons adjustments (**J-L**), or log-rank Mantel-Cox tests followed by Holm-Sidak's multiple comparisons adjustment (**M**). All statistical tests were two sided.
